# Lactylation-driven KRT19 promotes non-small cell lung cancer progression by suppressing cellular senescence

**DOI:** 10.1186/s13046-025-03602-5

**Published:** 2025-12-04

**Authors:** Cai Zhang, Yue Du, Yangyang Ji, Xiaoxiao Ye, Jingyao Lian, Haonan Zhou, Zihan Gao, Huiping Xu, Yuehan Tang, Yanhong Fan, Lu Zheng

**Affiliations:** 1 Department of Clinical Laboratory, The First Affiliated Hospital of Zhengzhou University, Zhengzhou, 450052 China; 2 Department of Clinical Laboratory Sciences, The First Clinical Medical College of Zhengzhou University, Zhengzhou, 450052 China; 3 Department of Blood Transfusion, The First Affiliated Hospital of Zhengzhou University, Zhengzhou, 450052 China

**Keywords:** Non-small cell lung cancer, Lactylation, Cellular senescence, KRT19, P21, Ubiquitination

## Abstract

**Background:**

Cellular senescence provides a protective barrier against tumorigenesis. However, the detailed mechanisms underlying tumor cells bypass senescence to malignant progression of non-small cell lung cancer (NSCLC) are still poorly understood.

**Methods:**

In this study, we assessed the impact of KRT19 on NSCLC using xenograft tumor models, EdU, CCK8, colony formation and transwell assay. We performed chromatin immunoprecipitation sequencing and dual luciferase reporter assay to explore the mechanism through which H3K18 lactylation (H3K18la) mediated KRT19. The mechanism underlying KRT19 regulated p21-driven cellular senescence was explored by senescence-associated β-galactosidase staining, flow cytometry and further identified by RNA sequencing, mass spectrometry, immunofluorescence, co-immunoprecipitation and protein ubiquitination assay. The clinical significance of H3K18la/KRT19/p21 was determined by immunohistochemistry in human NSCLC specimens and bioinformatics analysis of TCGA database and Kaplan-Meier method. We evaluated the effects of KRT19 inhibition and anti-PD-1 on NSCLC growth and immune infiltration using xenograft tumor models, flow cytometry and CIBERSORT.

**Results:**

Our study revealed that elevated expression of KRT19 was correlated with poor prognosis of NSCLC patients and exhibited oncogenic activity in NSCLC. Mechanistically, lactate-derived H3K18la activated the transcription of KRT19 via directly binding to its promoter. KRT19 blocked the transcriptional activation of p21 by p53, alternatively, KRT19 also interacted with MYH9 to facilitate ubiquitination of p21 at K16. More significantly, blockade of KRT19 potently enhanced the cytotoxic function of tumor-infiltrating CD8^+^ T cells and synergistically repressed NSCLC progression when combining with anti-PD-1.

**Conclusion:**

Our study emphasizes the importance of lactylation-driven KRT19 for overriding senescence and promoting NSCLC progression, reinforcing the potential of combination therapy strategies with KRT19 inhibitors to yield favorable responses in patients with NSCLC.

**Graphical abstract:**

Lactate-derived H3K18 lactylation increases the expression of KRT19, which overrides p21-driven cellular senescence to promote NSCLC progression through inhibiting the transcriptional activation of p21 by p53 and facilitating MYH9-mediated ubiquitination of p21 at K16. Silencing of KRT19 potently induces senescence program in NSCLC cells and boosts anti-PD-1 immunotherapy efficiency by potentiating stronger antineoplastic responses of tumor-infiltrating cytotoxic CD8^+^ T cells.

**Graphical abstract:**

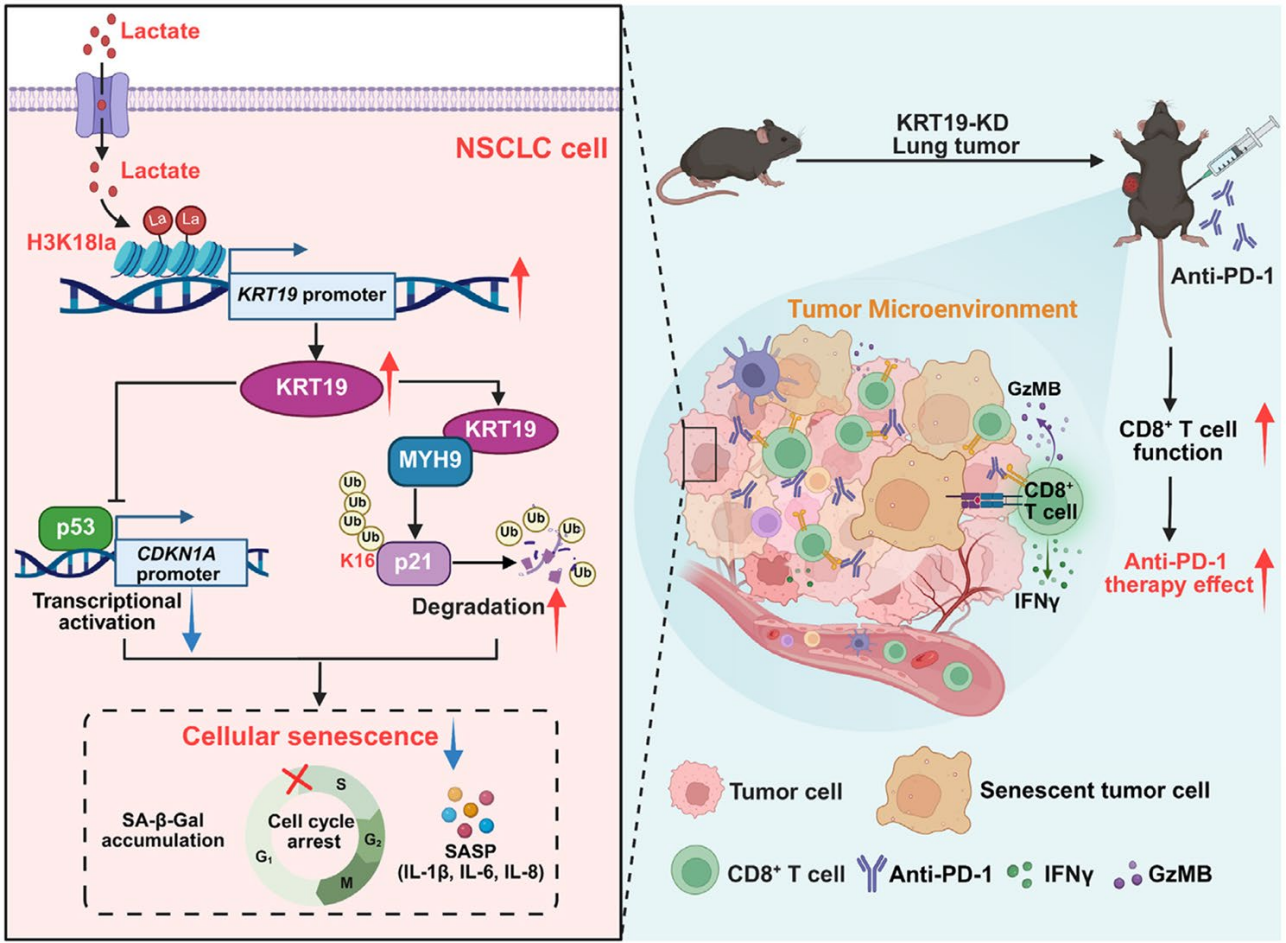

**Supplementary Information:**

The online version contains supplementary material available at 10.1186/s13046-025-03602-5.

## Background

Lung cancer is the most commonly diagnosed cancer and the leading cause of cancer-related mortality worldwide [1–3], and non-small cell lung cancer (NSCLC) accounts for approximately 85% of all diagnosed cases [4]. So far, limited approaches have been developed for early diagnosis, clinical therapies and prognosis of NSCLC [5–7], resulting in the grim reality of an incurable condition with the overall five-year survival rate only 26.4% [8]. Elucidating the molecular mechanisms underlying NSCLC development is essential for the evolution of novel therapeutic strategies for NSCLC [4].

Lactate, the end product of glycolysis, has been rediscovered as not only a major energy source, but also a vital signaling molecule serving non-metabolic functions [9]. *Zhang* et al. recently established lactate-derived histone lysine lactylation (Kla) as a novel post-translational modification (PTM) of lysine residues that directly stimulates transcription from chromatin to orchestrate the phenotypic shift from proinflammatory to tissue-repair state in macrophages [10]. Increasing evidence reveals the pivotal role of Kla in regulating tumor progression through histone modification [11–13] and non-histone protein modulation [14–17]. Histone H3 lysine 18 lactylation (H3K18la) enforces ubiquitin-specific protease 4 (USP4) upregulation to support glioblastoma stem cells maintenance and radiotherapy resistance by mediating stabilization and activity of Annexin A2 [13]. Elevated lactate levels could also promote p300-mediated H3K18la on the promoter of nicotinamide N-methyltransferase (NNMT) to induce resistance to epidermal growth factor receptor-tyrosine kinase inhibitors [18]. Extending our understanding of the pathogenic roles of H3K18la in NSCLC is of great significance for tumor prevention and accurate prognostic prediction.

As a member of the acidic type I cytokeratin family, keratin 19 (KRT19) is originally known to form the intermediate filament cytoskeleton and responsible for structural rigidity and multipurpose scaffolds [19]. KRT19 is abnormally expressed in various cancers including hepatocellular carcinoma [20], breast cancer [21, 22], colorectal cancer [23], papillary thyroid carcinoma [24], serous ovarian cystadenocarcinoma [25] and et al. KRT19 has been revealed to mediate cell cycle, cancer stem cell reprogramming by interacting with cellular molecules including receptors, adaptors, effectors and kinases. In hepatocellular carcinoma, KRT19 enhances the interaction of histone deacetylase 1 and REST corepressor 1 to increase the deacetylase activity of the corepressor of RE-1 silencing transcription factor (CoREST) complex, resulting in dedifferentiation and tumorigenesis [20]. In breast cancer, KRT19 could bind and stabilize human epidermal growth factor receptor 2 (HER2) via inhibition of ubiquitin-proteasome-mediated degradation of HER2 [26]. KRT19 also regulates cell cycle program by directly interacted with cyclin D3 [27]. Increasing evidence illustrates the clinical significance of KRT19 in lung cancer [28–30], extensive studies are urged to elucidate the role and underlying mechanism of KRT19 in NSCLC progression.

Cellular senescence, a stress-induced permanent cell-cycle arrest, undergoes phenotypic alterations including flattened and enlarged changes in cell morphology, production of a bioactive secretome termed senescence-associated secretory phenotype (SASP), macromolecular damage and metabolic reprogramming [31, 32]. SASP refers to the highly heterogeneous and dynamic secretory characteristics exhibited by senescent or stress-induced senescent cells, including the massive secretion of multiple pro-inflammatory cytokines (e.g. IL-6, IL-8 and IL-1α/β), chemokines (e.g. CXCL1, CCL2), growth factors (e.g. TGF-β, HGF) and non-protein molecules (e.g. extracellular vesicles, non-coding nucleic acids) [31]. It’s universally acknowledged that cellular senescence acts as a protective barrier against tumorigenesis upon numerous endogenous and exogenous stresses, such as nutrient deprivation, hypoxia and persistent DNA damage [33–36]. However, cancer cells could escape or bypass senescence to re-entry into the cell cycle and facilitate malignancy [37]. For example, RNA binding motif protein 4 (RBM4) competitively binds Liver kinase B1 (LKB1) and induces its ubiquitination and degradation in the nucleus to evade senescence and sustain cell growth of esophageal squamous cell carcinoma [38]. SET domain-containing protein 8 (SETD8) directly maintains H4K20me1 at the locus of cyclin dependent kinase inhibitor 1 A (CDKN1A) to repress p21-driven senescence in tumor cells independent of p53 [39]. In addition to directly influencing the behavior of tumor itself, senescent cells can also remodel the tumor immune microenvironment (TIME) via the SASP [40], activating immune surveillance to enhance therapeutic efficacy, or alternatively chronic inflammation that contributes to treatment failure and tumor recurrence [31, 41]. Deeper elucidation of the molecular dependencies and regulatory mechanisms underlying cellular senescence escape of NSCLC are crucial to invoke immune surveillance and harness senescence for tumor control.

Here, we revealed that lactate-derived H3K18la induced significant upregulation of KRT19, which exhibits oncogenic activity in NSCLC progression by overriding p21-dependent cellular senescence. Mechanistically, KRT19 inhibited p53 binding to the *CDKN1A* promoter, meanwhile, KRT19 bound to myosin heavy chain 9 (MYH9) to facilitate ubiquitination at lysine 16 (K16) of p21 and its subsequent degradation. Inspiringly, intervention of KRT19 in NSCLC cells enhanced anti-PD-1 immunotherapy efficiency by potentiating stronger antineoplastic responses of tumor-infiltrating cytotoxic CD8^+^ T cells (CTLs). Our study comprehensively characterizes the novel regulatory mechanism underlying H3K18la-driven KRT19 promotes NSCLC progression, yields promising insights into combination treatment strategies for patients with NSCLC.

## Methods

### Cell culture

PC-9, A549, NCI-H358, NCI-H1299 and NCI-H1944 cell lines were obtained from Cell Bank/Stem Cell Bank, Chinese Academy of Sciences. Lewis lung carcinoma (LLC), NCI-H1975 and HEK293T cell line were from Servicebio (Wuhan, China). All cell lines were authenticated by short tandem repeat profiling and certified as *Mycoplasma-free*. PC-9, A549, NCI-H358, NCI-H1944, NCI-H1299, NCI-H1975 and LLC cells were cultured in RPMI-1640 (L220KJ, BasalMedia) supplemented with 10% heat-inactivated fetal bovine serum (FBS, S660JY, BasalMedia) and penicillin-treptomycin (PS, C100C5, NCM Biotech). HEK293T cells were maintained in DMEM (L110KJ, BasalMedia) containing 10% FBS and PS. All cell lines were cultured less than 8 weeks after each thawing in 5% CO_2_ at 37 °C.

### Human specimens

NSCLC tissues and paracancerous normal tissues were obtained and the experiments were in accordance with relevant ethical regulations and approved by the Ethics Committee of the First Affiliated Hospital of Zhengzhou University (Zhengzhou, China).

### Mouse tumor models

BALB/c-nude mice (13001 A, Huafukang) or C57BL/6J mice (11001 A, Huafukang) were housed under the specific pathogen-free condition with a photoperiod (12-hour light/12-hour dark cycle). All experimental procedures were conducted in accordance with the Institutional Animal Care and Use Committee at the First Affiliated Hospital of Zhengzhou University.

For in vivo experiments, 1 × 10^6^ LLC cells or 1 × 10^6^ PC-9 cells were implanted subcutaneously into the upper left flank region of BALB/c-nude mice (5–6 weeks of age, female). When the tumor volume reached 50–100 mm^3^, mice were randomly divided into two groups and injected intratumorally with lactate (L7022, Sigma-Aldrich, 40 µL of 40 mM) or control saline. The mice were sacrificed to weigh the xenografted tumors after treatment. BALB/c-nude mice (5–6 weeks of age, female) were subcutaneously injected with 1 × 10^6^ PC-9 cells stably transduced with KRT19 shRNAs or control scramble shRNA, or 5 × 10^6^ A549 cells stably-overexpressing KRT19 or empty vector. Tumor size was measured daily or every 2 days until mice were sacrificed for further analysis. Tumor growth was monitored with the formula: V= (major axis) × (minor axis)^2^/2.

For the anti-PD-1 therapy, C57BL/6J mice (5 weeks of age, female) were implanted subcutaneously with 1 × 10^5^ Krt19-knockdown or control LLC cells and tumor volume was measured every 2 days. When tumor volume grew into 50–100 mm^3^, mice bearing Krt19-knockdown or control LLC cells were divided at random into four groups respectively, and then treated intraperitoneally with either 200 µg anti-PD-1 (S0B0594, Starter) or control IgG (S0B0788, Starter) every third day. Tumor volume was calculated as described above.

### Lentiviral vectors, plasmids and RNA interference

Lentiviral vectors encoding Flag-tagged KRT19 or shRNAs targeting KRT19 were generated by GeneChem (Shanghai, China). A549 and HEK293T cells were transduced with Flag-KRT19 lentivirus or lentiviral control vector. PC-9 and LLC cells were transduced with lentiviral particles containing KRT19 shRNAs or scramble shRNA respectively. All cell lines transduced with lentivirus were selected with puromycin (P8230, Solarbio) for 4 weeks to generate stable cell lines.

Plasmids were transfected with TRLIP DNA Transfection Reagent (KD0201, Kemix) and small interfering RNAs (siRNAs) were transfected using RNATransMate (E607402, Sangon) according to manufacturer’s instructions. HA-Ub plasmid was obtained from Addgene, Myc-p21 plasmid and MYH9-overexpressing plasmid were from Miaoling Bio (Wuhan, China). Myc-p21-K16R, Myc-p21-K75R, Myc-p21-K154R was generated by site-directed mutagenesis using Hieff Mut™ Targeted Mutagenesis Kit (11003ES10, Yeasen). Primers for mutation were listed in Table S1. All constructs were confirmed by DNA sequencing.

Small interfering RNAs targeting lactate dehydrogenase A (siLDHA) and LDHB (siLDHB) were from Sangon (Shanghai, China), siRNAs targeting p53, MYH9 and negative controls were obtained from GenePharma (Shanghai, China). See siRNA sequences in Table S2.

### Measurement of lactate concentration

Xenografted tumor tissues from saline- or lactate-treated mice were homogenized, and the intratumoral lactate content was tested with Lactic Acid Assay Kit (BC5340, Solarbio) following the manufacturer’s specifications.

### Cell viability assay

LLC and PC-9 cells were stimulated with lactate (40 mM) or vehicle. PC-9 and NCI-H1975 cells were treated with glycolysis inhibitor oxamate (HY-W013032A, MCE, 10 mM) and 2-Deoxy-D-glucose (2-DG; B1027, APExBIO, 20 mM) or vehicle, or transfected with siLDHA and siLDHB or control siRNA. A549 cells were transduced with KRT19-overexpressing lentiviral particles or lentiviral control vector, PC-9 cells were transduced with KRT19 shRNAs or scramble shRNA. The cell viability of LLC, PC-9, NCI-H1975 and A549 cells were measured by Cell Counting Kit-8 (CCK8; GK10001, GLPBIO) assay. In brief, cells were subjected to various treatments and seeded in 96-well plates at a density of 1,000 cells per well. At each time point, culture medium was removed, and 100 µL fresh medium containing 10 µL CCK8 reagent was added to per well, followed by 2-hour incubation in 5% CO2 at 37 °C. Then, the absorbance (450 nm) was measured with a microplate reader (51119080, Thermo Multiskan FC).

### EdU assay

For lactate treatment, LLC and PC-9 cells were stimulated with lactate (40 mM) or vehicle for 24 h. To investigate the impact of glycolysis inhibition on the NSCLC proliferation, PC-9 and NCI-H1975 cells were treated with 2-DG (20 mM), oxamate (10 mM) or vehicle for 24 h, or transfected with siLDHA and siLDHB or control siRNA. An equal number of LLC, PC-9 or NCI-H1975 cells under different treatments were seeded in 48-well plates for 24 h and incubated with 10 µM EdU reagent for another 2 h. Then, the cells were conducted with BeyoClick™ EdU Cell Proliferation Kit with AF488 (C0071S, Beyotime) according to manufacturer’s instruction. Cells were visualized by a microscope (DMI3000B, Leica) and the proportion of EdU positive cells was quantified with Image J (version 1.53q). To identify the effect of KRT19 on the NSCLC proliferation in vitro, A549 cells were transduced with KRT19-overexpressing lentivirus or lentiviral control vector, PC-9 were transduced with KRT19 shRNAs or scramble shRNA. EdU positive cells in different groups were detected with BeyoClick™ EdU Cell Proliferation Kit with AF488 (C0071S, Beyotime) as above.

### Colony formation assay

A549 cells with stable KRT19 overexpression or PC-9 cells with stable KRT19 knockdown were seeded in 6-well plates at a density of 5,000 cells per well and incubated in 5% CO2 at 37 ℃ with a fresh medium change every 2–3 days. Colonies were fixed with 4% paraformaldehyde (G1101, Servicebio) and stained with crystal violet (G1014, Servicebio). Cells were visualized by a microscope (DMI3000B, Leica) and the colony numbers were quantified by Image J (version 1.53q).

### Transwell assay

2 × 10^4^ stable KRT19 overexpression A549 cells or 4 × 10^4^ stable KRT19 knockdown PC-9 cells and equal number of corresponding control cells were suspended in 100 µL culture medium containing 1% FBS. Then, cells were inoculated on the upper chambers of a 24-well culture insert with permeable membrane (CLS3422, Corning), and the lower chambers were filled with 20% FBS medium as a chemoattractant. After cultured in a humidified incubator at 37 °C with 5% CO_2_ for 48 h, cells on the inside of the Transwell inserts were gently removed, and the cells on the lower surface were stained with crystal violet (G1014, Servicebio). The migrated cells were imaged with a microscope (DMI3000B, Leica) and quantified by Image J (version 1.53q).

### RNA extraction, cDNA synthesis and real-time PCR (RT-PCR)

Total RNA was extracted with TRIzol reagent (CW0580, CWBIO) based on the manufacturer’s specification, and cDNA was generated using the All-in-One Script RTpremix (MR0502, Kermey). RT-PCR was conducted with 2×SYBR Green qPCR Premix (MS0601, Kermey). The data were read using a Bio-Rad RT-PCR system (Roche LightCycler^®^ 480 II) and normalized to *ACTB* or *Actb* using ΔΔCt. The primers were described in Table S1.

### Chromatin Immunoprecipitation assay

ChIP-seq was performed on NCI-H1299 cells by Bioyigene Biotechnology Co.,Ltd. (Wuhan, China). In brief, NCI-H1299 cells were cross-linked with formaldehyde (1%, vol/vol) for 10 min, lysed, and dissociated by sonication. Then, cell lysis was immunoprecipitated with ChIP-grade anti-H3K18la (PTM-1427RM, PTM BIO) at 4 °C overnight, and the input sample was served as control. DNA was extracted by the phenolchloroform method. High-throughput DNA sequencing library preparation was conducted using the VAHTS Universal DNA Library Prep Kit for Illumina V3 (ND607, Vazyme). Library products with a size range of 200–500 bp were enriched, quantified, and sequenced on a Novaseq 6000 sequencer (Illumina) with PE150 model.

### ChIP-PCR assay

A549 or PC-9 cells subjected to different treatments were cross-linked with formaldehyde (1%, vol/vol), lysed and sonicated. Then, cell lysis was immunoprecipitated with ChIP-grade anti-H3K18la (PTM-1427RM, PTM BIO) or anti-p53 antibody (10442-1-AP, Proteintech) overnight at 4 °C, followed by washing, elution and cross-link reversal using the ChIP Assay Kit (P2078, Beyotime). Finally, the eluted DNA was extracted and tested using a Bio-Rad RT-PCR system (Roche LightCycler^®^ 480 II). Fold enrichment was calculated as a percentage of input chromatin. See primers in Table S1.

### Dual luciferase reporter assay

The *KRT19* promoter sequence (−448 bp to 163 bp) relative to the transcription start site was amplified using PCR and inserted into the pGL3-basic vector (PGL3-*KRT19* vector). A549 and PC-9 cells were co-transfected with pGL3-basic or pGL3-*KRT19*-Luc and pRL-TK (E2241, Promega) for 48 h. Then, A549 cells were incubated with lactate (40 mM) or vehicle for 16 h, PC-9 cells were treated with 2-DG (20 mM), oxamate (10 mM) or vehicle for 16 h. The luciferase activity was tested using the DLR Assay System (E1910, Promega) with BioTek Synergy H1, and the ratio of Firefly to Renilla was quantified.

### RNA sequencing and analysis

A549 cells were transduced with KRT19-overexpressing lentivirus or lentiviral empty vector, PC-9 cells were transduced with KRT19 shRNA or scramble shRNA. Total RNA was isolated using TRIzol reagent (CW0580, CWBIO). The GeneChem (Shanghai, China) and Sangon (Shanghai, China) carried out RNA-Seq, data cleaning, and standardization. Kyoto Encyclopedia of Genes and Genomes (KEGG) pathway and Gene Set Enrichment Analysis (GSEA) were performed using R software (version 4.3.1). The differentially expressed genes between KRT19-overexpressing A549 and control A549 cells were defined by *p* < 0.05. These differentially expressed genes were intersected with senescence-associated genes from the CellAge database to identify overlapping candidates, visualized via hierarchical clustering heatmap (Z-score normalized expression).

### Bioinformatics analysis

Transcriptome data of NSCLC patients were obtained from The Cancer Genome Atlas Program (TCGA) database, and lung adenocarcinoma (LUAD) data set includes 513 tumor samples and 58 normal samples, lung squamous carcinoma (LUSC) data set consists of 496 tumor samples and 51 normal samples. R software (version 4.3.1) was used for bioinformatics analysis. Differential expression analysis was conducted with |log_2_FC| >1 and *p* < 0.05. Survival curves were generated with the Kaplan-Meier method and were evaluated by the log-rank test using Kaplan-Meier Plotter.

CIBERSORT, a metagene analysis tool, was used to determine the infiltration scores of 22 distinct immune cell types within each tumor sample. The association between *KRT19* expression and immune cell infiltration scores was calculated using R software (version 4.3.1). In addition, correlations between *KRT19* and various immune cell infiltrations were explored using the TIMER2.0 website.

### Immunoblot

Tissues and cells were lysed with RIPA buffer (G2002, Servicebio) containing protease inhibitor (G2007, Servicebio) and 1 mM PMSF (G2008, Servicebio). Protein extracts were quantified with the BCA Protein Assay Kit (WB6501, NCM Biotech), and immunoblotted with primary antibodies against H3K18la (PTM-1406RM, PTM BIO), Histone H3 (17168-1-AP, Proteintech), KRT19 (10712-1-AP, Proteintech), MYH9 (11128-1-AP, Proteintech), p53 (10442-1-AP, Proteintech), p21 (10355-1-AP, Proteintech), CDK1 (ab133327, Abcam), CDK6 (CY5835, Abways), Cyclin D1 (CY5404, Abways), LDHA (CY5348, Abways), LDHB (sc-100775, Santa Cruz), Ubiquitin (sc-166553, Santa Cruz), α-Tubulin (66031-1-Ig, Proteintech), Flag tag (66008-4-Ig, Proteintech), HA tag (51064-2-AP, Proteintech), Myc tag (AB0001, Abways) and appropriated secondary horseradish peroxidase-labeled antibodies. Indicated protein was detected with Enhanced chemiluminescence (SQ201, Epizyme) and imaged on Azure Biosystems C300.

### Co-immunoprecipitation (Co-IP)

For immunoprecipitation assays, cells were transduced with lentiviral particles and transfected with plasmids or small interfering RNAs as required. Cell extracts were lysed with IP Lysis Buffer (G2038, Servicebio) containing protease inhibitor (G2007, Servicebio) and 1 mM PMSF (G2008, Servicebio) for 30 min at 4 °C. After centrifugation at 12,000×g for 10 min at 4 °C, the supernatant was collected and rotated with protein A + G agarose (P0255, Beyotime) for 1 h at 4 °C, and centrifuged at 3,000×g for 5 min at 4 °C. Then, the supernatant was harvested and incubated with specific antibodies or control IgG at 4 °C. After overnight incubation, the samples were rotated with protein A + G agarose (P0255, Beyotime) for 3 h at 4 °C, and the agarose was collected, washed three times with IP wash buffer and eluted with SDS-PAGE loading buffer for immunoblot analysis.

### Mass spectrometry

To identify the proteins interacted with KRT19, total extracts of A549 cells with stable Flag-KRT19 overexpression were immunoprecipitated with Flag tag antibody (66008-4-Ig, Proteintech) or control IgG as previously described, and subjected to SDS-PAGE. Then, the gels were stained using a Fast Silver Stain kit (P0017S, Beyotime) according to the manufacturer’s instructions. Subsequently, the silver-stained bands of interest were excised and processed for mass spectrometric analysis using an Ultimate 3000 nano ultra-performance liquid chromatography-tandem Q Exactive plus mass spectrometry system (LuMing Biotech, Shanghai, China).

### Senescence-associated β-galactosidase (SA-β-gal) staining

A549 cells transduced with Flag-KRT19 lentivirus or lentiviral control vector, or transfected with MYH9-overexpressing plasmid or empty vector were treated with 50 µM hydrogen peroxide (H_2_O_2_) for 24 h. PC-9 cells were transduced with lentiviral particles containing KRT19 shRNAs or scramble shRNA, or transfected with MYH9 siRNA or control siRNA. Then, cells were incubated with fresh β-gal staining solution at 37 °C for 20 h, and SA-β-gal activity was measured by SA-β-Gal Staining Kit (C0602, Beyotime) referring to the manufacturer’s protocol. For rescue assays, PC-9 cells were transduced with either shKRT19 lentiviral particles alone or co-transfected with MYH9-overexpressing plasmid for 48 h and then subjected to SA-β-gal staining. The SA-β-gal positive cells were captured using a microscope (DMI3000B, Leica) and quantified by Image J (version 1.53q).

### Histology/Immunohistochemistry (IHC)

Paraffin-embedded tissue samples were performed with hematoxylin and eosin (H&E) and IHC. IHC was conducted according to manufacturing protocol using primary antibodies against H3K18la (PTM-1406RM, PTM BIO), KRT19 (10712-1-AP, Proteintech), p21 (10355-1-AP, Proteintech) and Ki-67 (GB111499, Servicebio). The immunohistochemical reactivity of H3K18la, KRT19 and p21 was assessed by two pathologists independently as follows: multiplication of the percentage of positive cells and the immunostaining intensity (0-, no reactivity; 1-, weak; 2-, moderate; 3-, strong), which resulted in an immunoreactive score (IRS) of 0–300.

### Immunofluorescence

A549 and HEK293T cells co-transfected with Flag-KRT19 lentivirus and MYH9-overexpressing plasmid or control vector were seeded on coverslips in 24-well plates and cultured for 24 h. Culture medium was removed, cells were washed with PBS and fixed in 4% paraformaldehyde (G1101, Servicebio) for 15 min, followed by cell permeabilization with 0.1% Triton X-100 (G3068, Servicebio) for 10 min. Then, A549 and HEK293T cells were washed with PBS and blocked by 5% bovine serum albumin (BSA; MB0101, Kermey) for 1 h, incubated with anti-Flag and anti-MYH9 overnight at 4 °C and secondary antibodies labeled with AF488/594 (AB0141/AB0152, Abways) for 1 h. Finally, cell nuclear was staining with DAPI (MD0139, Kermey) for 10 min. The images were captured by Axio Scope A1 microscopy (Zeiss) and the co-localization analysis of Flag-KRT19 and MYH9 was determined with Image J (version 1.53q).

Immunofluorescence staining of LLC tumor tissues was performed as standard protocol with primary antibody against CD8 (GB15068, Servicebio). The images were captured by Axio Scope A1 microscopy (Zeiss). Number of tumor-infiltrating CD8^+^ T cells was calculated with Image J (version 1.53q).

### Flow cytometry

For cell cycle analysis, A549 cells with stable KRT19 overexpression or PC-9 cells with stable KRT19 knockdown and control cells were washed with PBS and fixed in 70% ethanol at 4 °C overnight. Then, the cells were washed with PBS and stained with propidium iodide (PI, C0080, Solarbio) containing RNase A (R1030, Solarbio) for 15 min and analyzed with BD FACSVerse™.

For measurement of tumor-infiltrating CTLs function, tumor tissues collected from LLC-bearing C57/BL6J mice were minced, digested with 200 µg/mL collagenase IV (17104019, Thermo Fisher Scientific) and 40 µg/mL DNase I (10104159001, Roche) for 90 min at 37 ℃, and filtered with cell strainers (BS-40-CS, Biosharp). Then, tumor-infiltrating leukocytes were separated from single cell suspensions with a Percoll gradient (17–0891-01, Cytiva), incubated with Cell Stimulation Cocktail (423303, BioLegend) at 37 ℃ for 4 h, and stained with fluorescent antibodies against CD3 (100325, BioLegend) and CD8 (100711, BioLegend) for 30 min at 4 ℃ in the dark. For intracellular staining, CD8^+^ T cell samples isolated from tumor tissues were fixed and permeabilized with Cytofix/Cytoperm Solution (554715, BD), followed by staining with fluorescent antibodies against IFN-γ (505806, BioLegend) and Granzyme B (GzMB, 372207, BioLegend). Data were acquired on a BD FACSVerse™ system and analyzed with FlowJo.

### Statistical analysis

Data are presented as mean ± SEM. Statistical significance was calculated using paired Student’s *t*-test or unpaired Student’s *t*-test and for variances by ANOVA using GraphPad Prism (version 9.0.0). Statistical *p*-value < 0.05 was considered statistically significant.

## Results

### Lactate directly accelerates NSCLC progression

**Fig. 1:**
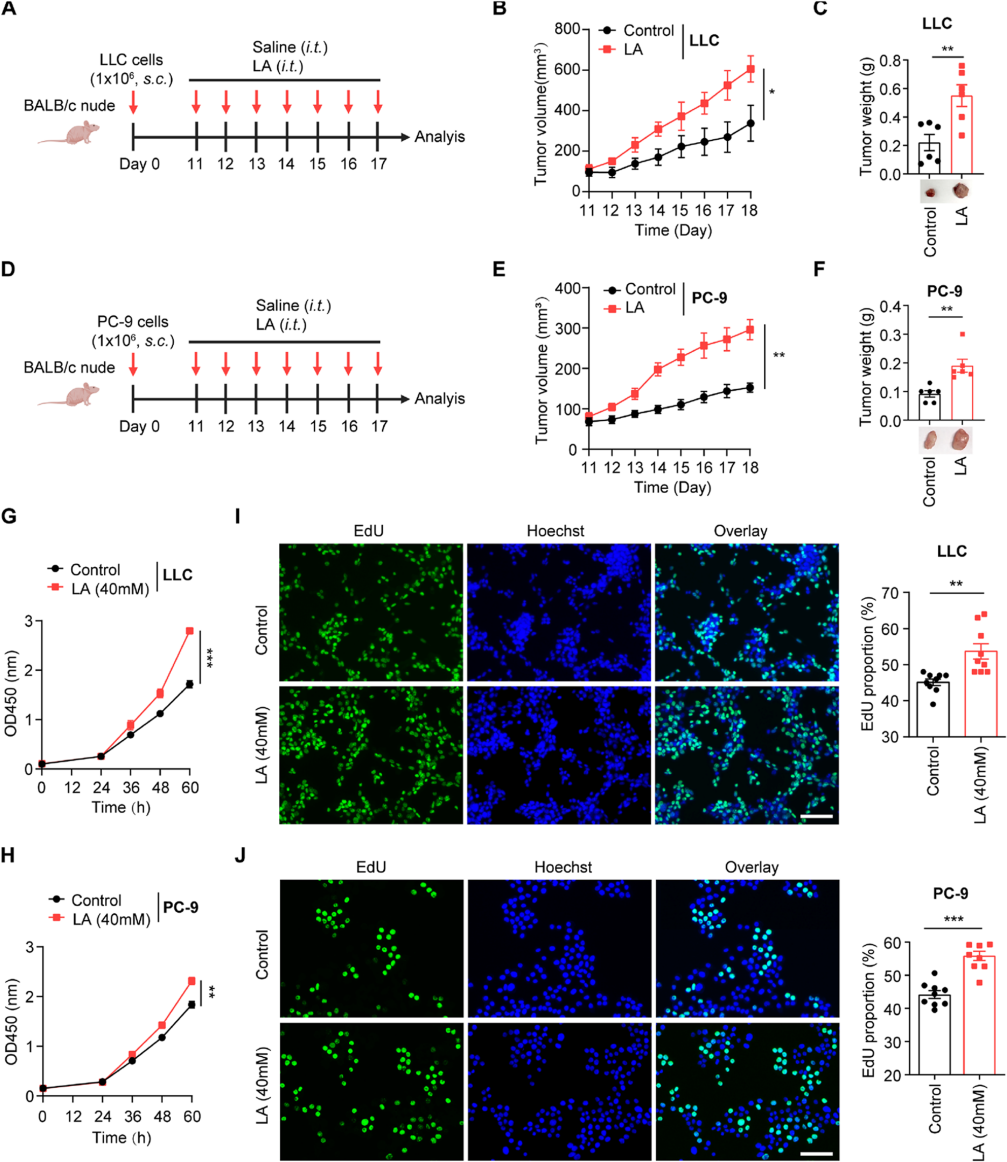
Lactate directly accelerates NSCLC progression. **A**-**F** BALB/c-nude mice (5–6 weeks of age, female) were subcutaneously (*s.c.*) injected with 1 × 10^6^ LLC or 1 × 10^6^ PC-9 cells and intratumorally (*i.t.*) treated with either lactate (40 µL of 40 mM, LA group) or saline (Control group) daily when the tumor tissues reached 50 mm^3^. Schematic depicting of experimental setup (A, D). Tumor growth curves (B, E), tumor weights and representative images of tumors (C, F) were shown (*n* = 6) **G-H** Cell viability of LLC (G) and PC-9 cells (H) treated with lactate (40 mM, LA group) or vehicle (Control group) for indicated time was determined by CCK8 assay **I-J** Proliferation of LLC (I) and PC-9 cells (J) treated with lactate (40 mM, LA group) or vehicle (Control group) for 24 h was assessed by EdU assay, representative images (left) and quantification of EdU-positive cells (right) were shown. Green, EdU; blue, Hoechst. Scale bar, 50 μm (3 technical replicate wells, 3 fields per well). Data are shown as mean ± S.E.M. and determined by Student’s *t*-test (B-C, E-J). *p < 0.05; **p < 0.01; ***p < 0.001. The experiments (G-J) were repeated three times

Excessive lactate within tumors facilitates the tumor cell biology through multiple pathways [42]. We have identified an aberrant increase in glycolytic capacity and elevated lactate content in NSCLC tissues [43]. To explore the significance of lactate in NSCLC progression, we subcutaneously injected LLC or PC-9 cells into the BALB/c-nude mice and treated with lactate or saline. Compared with the control group, mice treated with lactate showed a significantly larger tumor burden (Fig. 1A-F). Moreover, intratumoral lactate level was positively correlated with tumor volume in both LLC- and PC-9-bearing mice (Figure S1A-D).

Next, we substantiated the effect of lactate in vitro. CCK8 assay found that lactate significantly accelerated the proliferation rate of LLC and PC-9 cells (Fig. 1G-H). Consistently, EdU assay also showed a marked increase in the number of EdU-positive LLC and PC-9 cells after lactate treatment compared to the control group (Fig. 1I-J). As a key enzyme of glycolysis, lactate dehydrogenase consists of two major subunits LDHA and LDHB. Simultaneous silencing of LDHA and LDHB intrinsically suppressed PC-9 and NCI-H1975 cell proliferation (Figure S1E-H). Consistently, glycolysis inhibitor 2-DG and oxamate also showed a profound inhibitory effect on the proliferative capacity of PC-9 and NCI-H1975 cells (Figure S1I-L).

Altogether, these findings further validate the important role of lactate in promoting NSCLC progression.

### KRT19 exerts oncogenic activity in NSCLC

**Fig. 2:**
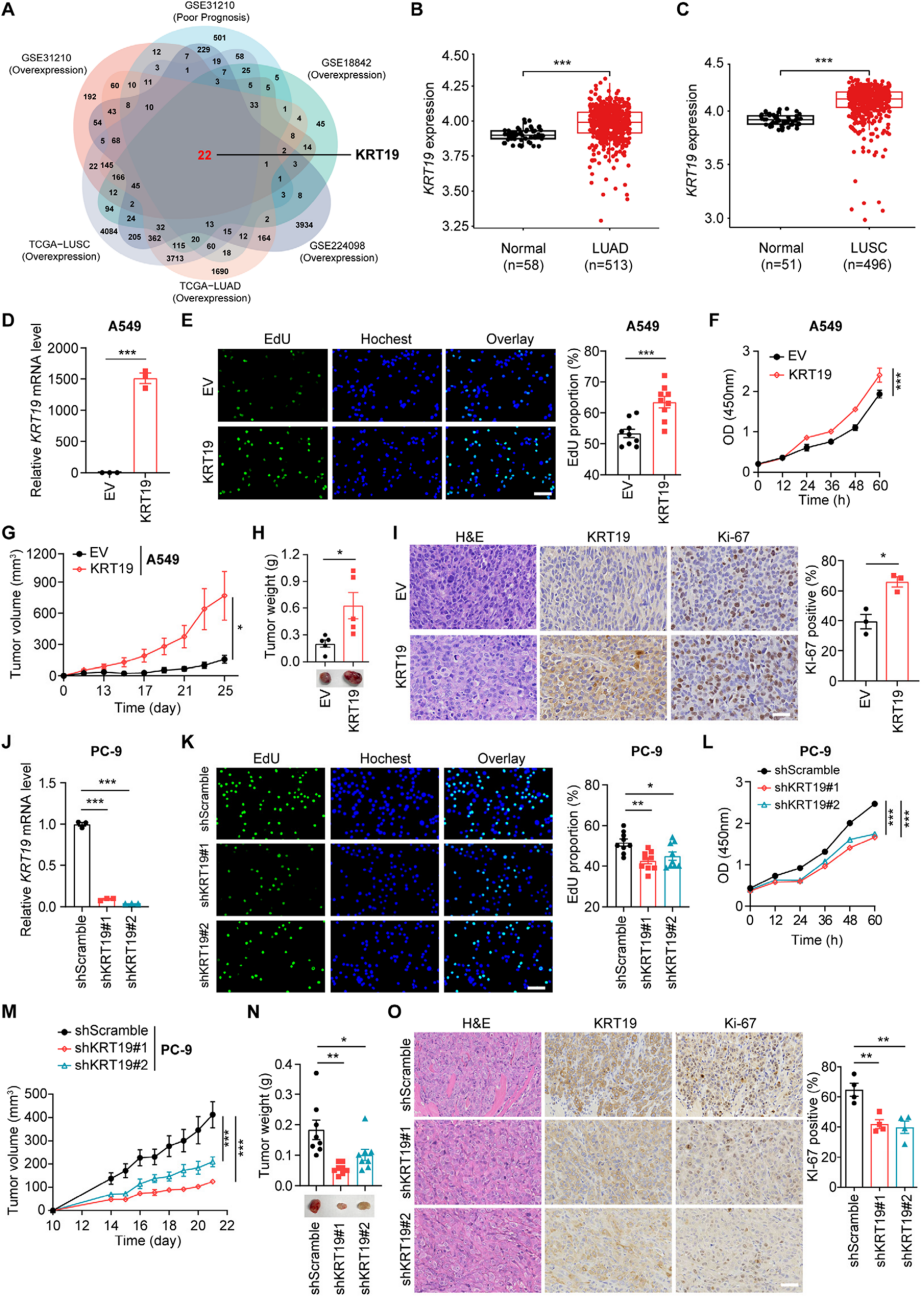
KRT19 exerts oncogenic activity in NSCLC **A** Venn diagram of an integrated analysis based on GSE31210, GSE18842, GSE224098, TCGA-LUAD and TCGA-LUSC **B-C** *KRT19* mRNA expression levels in LUAD (B) and LUSC (C) samples obtained from TCGA database were analyzed using R software (version 4.3.1) **D-I** A549 cells were transduced with KRT19-overexpressing lentiviral vector (KRT19 group) or control empty vector (EV group) **D** *KRT19* mRNA expression in A549 cells (EV, KRT19) was measured by RT-PCR **E** Proliferation of A549 cells (EV, KRT19) was assessed by EdU assay, representative images (left) and quantification of EdU-positive cells (right) were shown. Green, EdU; blue, Hoechst. Scale bar, 50 μm (3 technical replicate wells, 3 fields per well) **F** Cell viability of A549 cells (EV, KRT19) was determined by the CCK8 assay **G-I** BALB/c-nude mice (5 weeks of age, female) were subcutaneously implanted with 5 × 10^6^ A549 cells (EV, KRT19). Tumor growth curves (G), tumor weights and representative images of tumors (H) were shown (*n* = 5). (I) Representative images of H&E and IHC staining with KRT19 and Ki-67 within tumor tissues were shown (*n* = 3). Scale bar, 50 μm **J-O** PC-9 cells were transduced with scramble shRNA lentivirus (shScramble) or KRT19 shRNA lentivirus (shKRT19#1, shKRT19#2) **J** *KRT19* mRNA expression in PC-9 cells (shScramble, shKRT19#1, shKRT19#2) was determined by RT-PCR **K** Proliferation of PC-9 cells (shScramble, shKRT19#1, shKRT19#2) was assessed by EdU assay. Green, EdU; blue, Hoechst. Scale bar, 50 μm (3 technical replicate wells, 3 fields per well) **L** Cell viability of different groups of PC-9 cells was determined by the CCK8 assay **M-O** BALB/c-nude mice (5 weeks of age, female) were subcutaneously implanted with 1 × 10^6^ PC-9 cells (shScramble, shKRT19#1, shKRT19#2). Tumor growth curves (M), tumor weights and representative image of tumors (N) were shown (*n* = 8). (O) Representative images of H&E and IHC staining with KRT19 and Ki-67 within PC-9 tumor tissues were shown (*n* = 4). Scale bar, 50 μm. Data are shown as mean ± S.E.M. and analyzed by Student’s *t*-test. *p < 0.05; **p < 0.01; ***p < 0.001. The experiments (D-F, J-K) were repeated three times

In this section, we performed an integrated analysis of multi-cohort datasets (GSE31210, GSE18842, GSE224098, TCGA-LUAD, TCGA-LUSC) and comprehensively revealed 22 differentially overexpressed candidate genes in NSCLC, which were also positively correlated with poor prognosis and remarkably upregulated in the context of lactate accumulation (Fig. 2A). Of the 22 identified genes, KRT19 was the only member of the keratin family that met the criteria for the integrated analysis. Notably, aberrant KRT19 expression has been implicated in tumor proliferation, metastasis and survival in hepatocellular carcinoma [20], breast cancer [21, 22], colorectal cancer [23], and is closely associated with the prognosis of various tumors [44]. Based on TCGA database, we verified that NSCLC exhibited higher KRT19 expression than control group (Fig. 2B-C). To investigate the biological function of KRT19 in NSCLC cells, we stably-overexpressed KRT19 in A549 cells using lentiviral particles (Fig. 2D**)**. EdU, CCK8 and colony formation assay showed that KRT19 overexpression significantly enhanced the proliferation and cluster formation of A549 cells in vitro (Fig. 2E-F, Figure S2A). Furthermore, we subcutaneously injected empty vector (EV group) or KRT19-overexpressing (KRT19 group) A549 cells into the BALB/c-nude mice. Compared with the EV group, mice received KRT19-overexpressing A549 cells showed increased tumor growth rate and tumor weight (Fig. 2G-H), which also exhibited higher Ki-67 levels in tumor tissues determined by immunohistochemical staining (Fig. 2I). In addition, KRT19 overexpression accelerated the migration of A549 cells as judged by transwell assay (Figure S2B). On the other side, we also knocked down KRT19 using shRNA lentiviral particles in PC-9 cells (Fig. 2J). Compared with the corresponding control group, silencing of KRT19 intrinsically suppressed the proliferation and migration of PC-9 cells in vitro and in vivo (Fig. 2K-O, Figure S2C-D). Collectively, these data identified the oncogenic role of KRT19 in NSCLC progression.

### H3K18la binds to the *KRT19* promoter to upregulate KRT19 expression in NSCLC cells

**Fig. 3:**
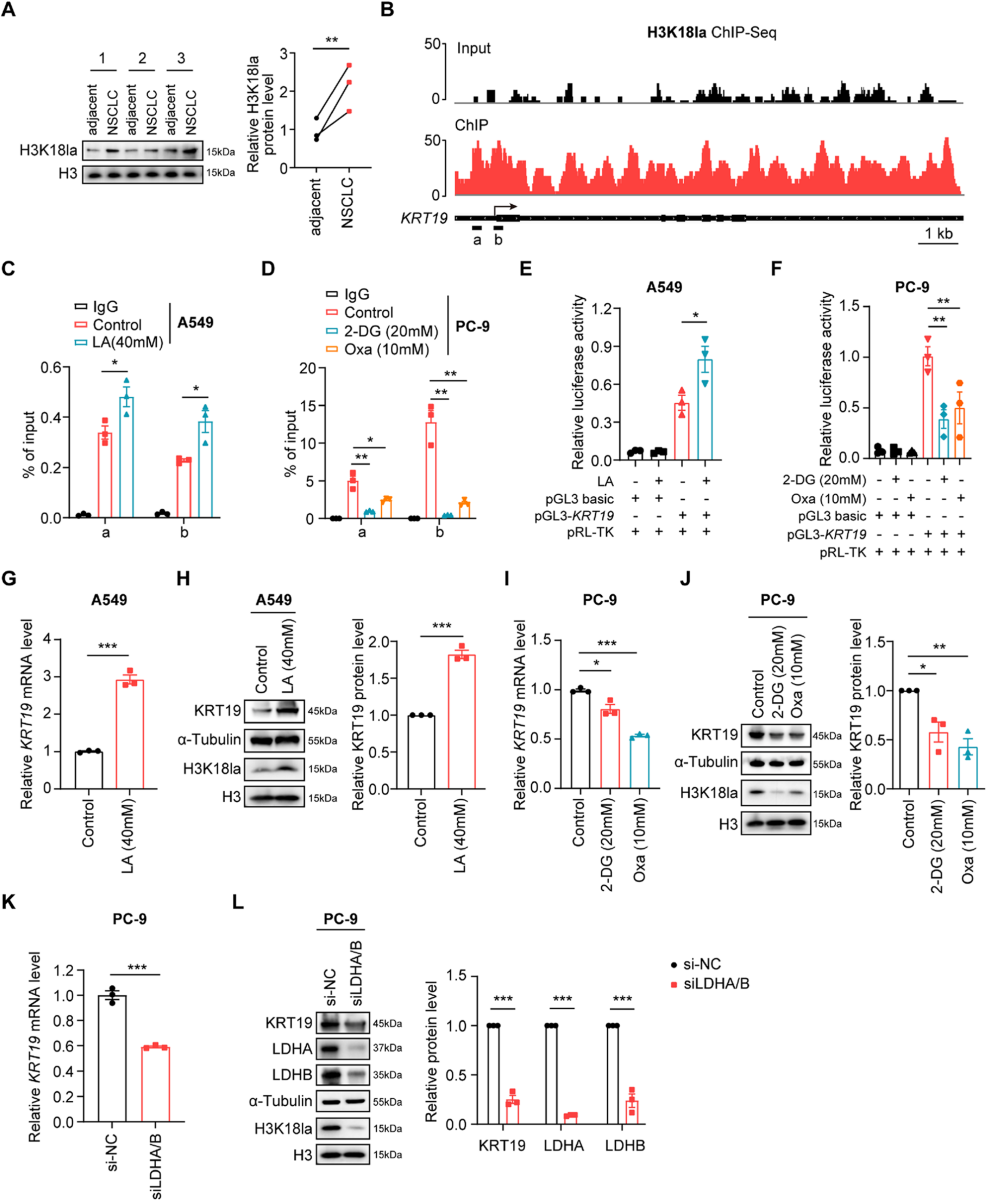
H3K18la binds to the *KRT19* promoter to upregulate KRT19 expression in NSCLC cells **A** The protein expression of H3K18la in clinical NSCLC tissues and paired normal tissues (*n* = 3) were determined by immunoblot **B** ChIP-seq with anti-H3K18la was performed on NCI-H1299 cells, and input was served as control. IGV tracks for KRT19 from ChIP-seq data (GSE245990) were shown **C** A549 cells were incubated with lactate (40 mM, LA group) or vehicle (Control group) for 16 h, the binding of H3K18la to the *KRT19* promoter regions (a, b) in control and lactate-treated A549 cells was determined by ChIP-PCR **D** PC-9 cells were treated with 2-DG (20 mM, 2-DG group), oxamate (10 mM, Oxa group) or vehicle (Control group) for 16 h. The enrichment of H3K18la in the *KRT19* promoter regions (a, b) in different groups of PC-9 cells was measured by ChIP-PCR **E** A549 cells were co-transfected with pRL-TK and pGL3-basic or pGL3-*KRT19-*Luc plasmids for 48 h, and then stimulated with or without lactate (40 mM, LA group) for another 16 h, the luciferase activities were determined **F** PC-9 cells were co-transfected with pRL-TK and pGL3-basic or pGL3-*KRT19-*Luc plasmids for 48 h, and treated with or without 2-DG (20 mM) or oxamate (10 mM, Oxa) for another 16 h. The luciferase activities were determined **G-H** A549 cells were incubated with lactate (40 mM, LA group) or vehicle (Control group) for 24 h, KRT19 mRNA (G) and protein (H) expression was determined by RT-PCR and immunoblot, respectively **I-J** KRT19 mRNA (I) and protein (J) levels in PC-9 cells treated with 2-DG (20 mM, 2-DG group), oxamate (10 mM, Oxa group) or vehicle (Control group) for 24 h were measured **K-L** PC-9 cells were transfected with siLDHA and siLDHB (siLDHA/B group) or control siRNA (si-NC group) **K** *KRT19* mRNA expression in PC-9 cells (si-NC, siLDHA/B) was determined by RT-PCR **L** KRT19, LDHA, LDHB and H3K18la protein levels in different groups of PC-9 cells were measured using immunoblot. Data are shown as mean ± S.E.M. and analyzed by Student’s *t*-test (A, C-L). *p < 0.05; **p < 0.01; ***p < 0.001. All experiments were repeated two (B) or three times (C-L)

Given that KRT19 was remarkably upregulated in the context of lactate accumulation based on integrated multi-cohort dataset analysis (Fig. 2A), we next investigated the underlying molecular mechanism. Histone lactylation is a novel epigenetic modification that uses lactate as a substrate and directly promotes gene transcription on chromatin, among which H3K18la plays important roles in tumor growth, metastasis and therapy resistance and has attracted considerable attention. Immunoblot analysis showed that clinical NSCLC tissues exhibited elevated H3K18la levels compared with paired adjacent normal tissues (Fig. 3A). Then, we performed ChIP-seq (GEO accession number: GSE245990) and the candidate genomic loci identified at *KRT19* showed that the binding of H3K18la to the *KRT19* promoter was enriched in NSCLC cells (Fig. 3B). Referring to the differential expression of H3K18la in human PC-9 and A549 cell lines (Figure S3A), we performed ChIP-PCR and found that the level of H3K18la at the *KRT19* promoter was significantly increased upon lactate stimulation in A549 cells, while treatment with glycolysis inhibitor 2-DG and oxamate dramatically blocked H3K18la binding to the *KRT19* promoter in PC-9 cells (Fig. 3C-D). Moreover, we constructed luciferase reporter vector containing *KRT19* promoter and further identified that lactate significantly enhanced the luciferase activity driven by the *KRT19* promoter, which was inhibited by 2-DG and oxamate (Fig. 3E-F). Consistently, lactate treatment significantly elevated KRT19 mRNA and protein level in NSCLC cells compared to control vehicle (Fig. 3G-H, Figure S3B). Glycolysis inhibition by 2-DG and oxamate or simultaneous silencing of LDHA and LDHB markedly reduced H3K18la levels, along with decreased KRT19 expression in NSCLC cells (Fig. 3I-L, Figure S3C).

Altogether, these findings demonstrate that lactate-derived H3K18la activates the transcription of KRT19 via directly binding to its promoter to potentiate NSCLC progression.

### KRT19 suppresses cell senescence to promote NSCLC growth

**Fig. 4:**
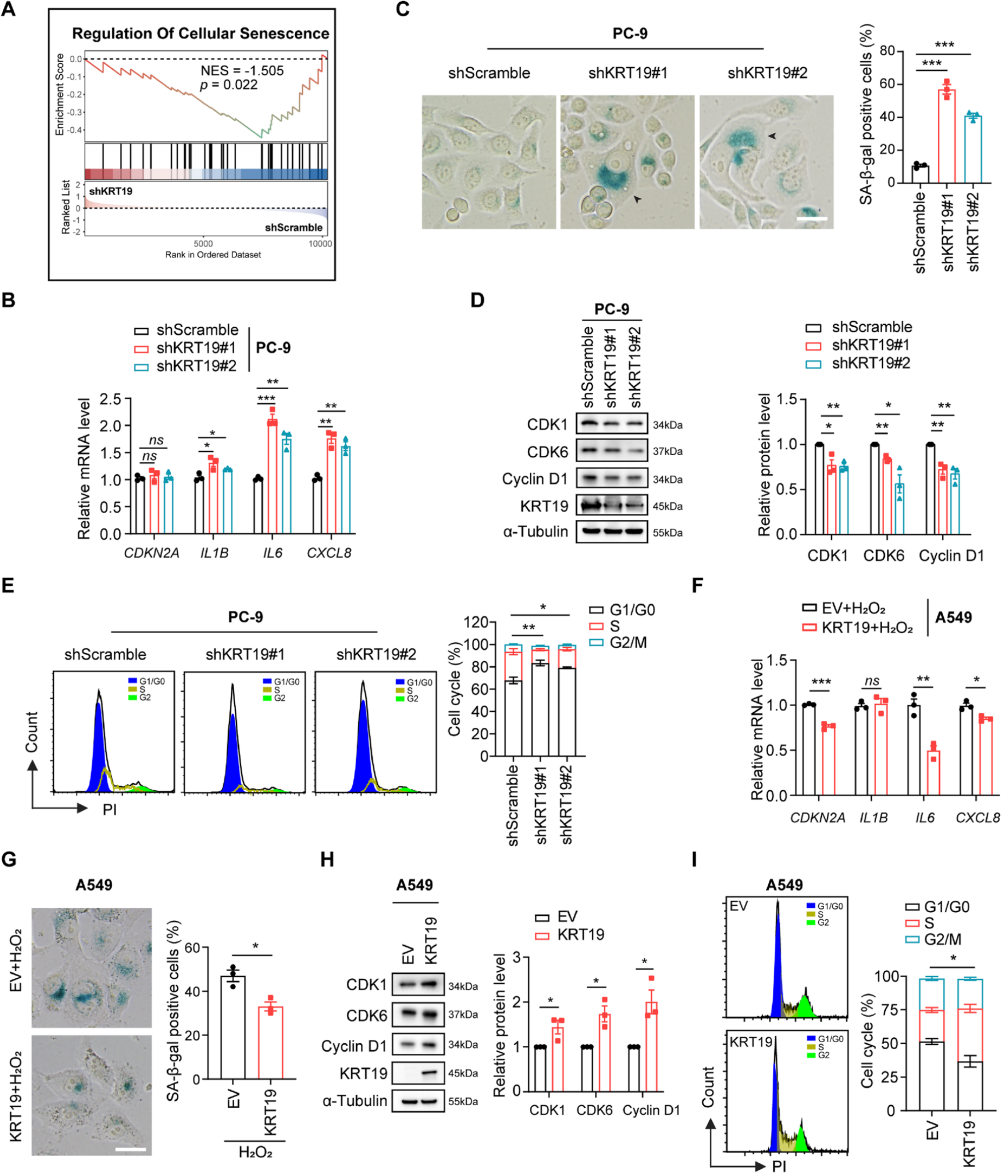
KRT19 suppresses cell senescence to promote NSCLC growth **A** RNA-seq (GSE299060) was performed in PC-9 cells transduced with scramble shRNA lentivirus (shScramble) or KRT19 shRNA lentivirus (shKRT19#1). GSEA plot evaluating cellular senescence in PC-9 cells (shScramble, shKRT19#1) **B** PC-9 cells were transduced with scramble shRNA lentivirus (shScramble) or KRT19 shRNA lentivirus (shKRT19#1, shKRT19#2). *CDKN2A*,* IL1B*,* IL6* and *CXCL8* mRNA expression in PC-9 cells (shScramble, shKRT19#1, shKRT19#2) was measured by RT-PCR **C** SA-β-gal accumulation in PC-9 cells (shScramble, shKRT19#1, shKRT19#2) was determined by SA-β-Gal Staining Kit. The arrow indicates the enlarged morphologic changes in senescent cells. Scale bar, 10 μm **D** CDK1, CDK6, Cyclin D1 and KRT19 protein levels in PC-9 cells (shScramble, shKRT19#1, shKRT19#2) were measured by immunoblot **E** Flow cytometric analysis of cell cycle in PC-9 cells (shScramble, shKRT19#1, shKRT19#2) using propidium iodide (PI) staining, and the percentage of cells in S phase was compared **F** A549 cells transduced with KRT19-overexpressing lentiviral vector (KRT19 group) or control empty vector (EV group) were treated with hydrogen peroxide (H_2_O_2_, 100 µM) for 24 h, *CDKN2A*,* IL1B*,* IL6* and *CXCL8* mRNA levels of A549 cells (EV, KRT19) were measured by RT-PCR **G** A549 cells transduced with KRT19-overexpressing lentiviral vector (KRT19 group) or control empty vector (EV group) were treated with hydrogen peroxide (H_2_O_2_, 50 µM) for 24 h. SA-β-gal staining of A549 cells (EV, KRT19) was shown. Scale bar, 10 μm **H** CDK1, CDK6, Cyclin D1 and KRT19 protein levels in A549 cells (EV, KRT19) were measured by immunoblot **I** Flow cytometric measurement of cell cycle in A549 cells (EV, KRT19) using propidium iodide (PI) staining, and the percentage of cells in S phase was compared. Data are shown as mean ± S.E.M. and analyzed by Student’s *t*-test (B-I). *p < 0.05; **p < 0.01; ***p < 0.001; ns, not significant. The experiments (B-I) were repeated three times

To further investigate how KRT19 regulates NSCLC progression, we analyzed the transcriptional alterations of KRT19-depleted PC-9 cells by the RNA-seq (GEO accession number: GSE299060). GSEA showed an aberrant activation of cellular senescence signal in PC-9 cells after KRT19 knockdown (Fig. 4A). Senescent cells display SASP through secreting pro-inflammatory factors, cytokines and chemokines [31, 38]. Both inhibiting KRT19 by 2-DG and oxamate or silencing of KRT19 using shRNAs led to enlarged morphologic changes, remarkably upregulated the SASP factors *IL1B*, *IL6* and *CXCL8* mRNA expression and facilitated the accumulation of senescence-associated β-galactosidase (SA-β-gal) in PC-9 cells, indicating a rise in the number of senescent cells (Fig. 4B-C, Figure S4). Cellular senescence suppresses tumorigenesis by stably arresting proliferation [38]. Immunoblot and flow cytometric analysis showed that KRT19 knockdown could significantly downregulate proliferation-related CDK1, CDK6 and Cyclin D1 protein expression, which resulted in cell cycle arrest in G1 phase (Fig. 4D-E), supporting the notion that KRT19 knockdown induced cellular senescence of NSCLC. On the other side, we also overexpressed KRT19 in A549 cells by lentiviral particles and performed RNA-seq (GEO accession number: GSE298926) in EV or KRT19-overexpressing A549 cells. GSEA and KEGG analysis ensured that overexpression of KRT19 led to the activation of cell cycle pathway (Figure S5). Consistently, RT-PCR and SA-β-gal staining assays showed lower *CDKN2A*, *IL6* and *CXCL8* mRNA expression and decreased SA-β-gal intensity in KRT19-overexpressing A549 cells compared with corresponding control under hydrogen peroxide stimuli (Fig. 4F-G). Moreover, KRT19 could remarkably upregulate CDK1, CDK6 and Cyclin D1 protein expression and promote cell cycle transition from G1 to S phase in A549 cells (Fig. 4H-I).

Taken together, our data indicated that KRT19 promotes NSCLC progression by overriding cellular senescence.

### KRT19 reduces p53-dependent transcription of p21 and impairs p21 protein stability

**Fig. 5:**
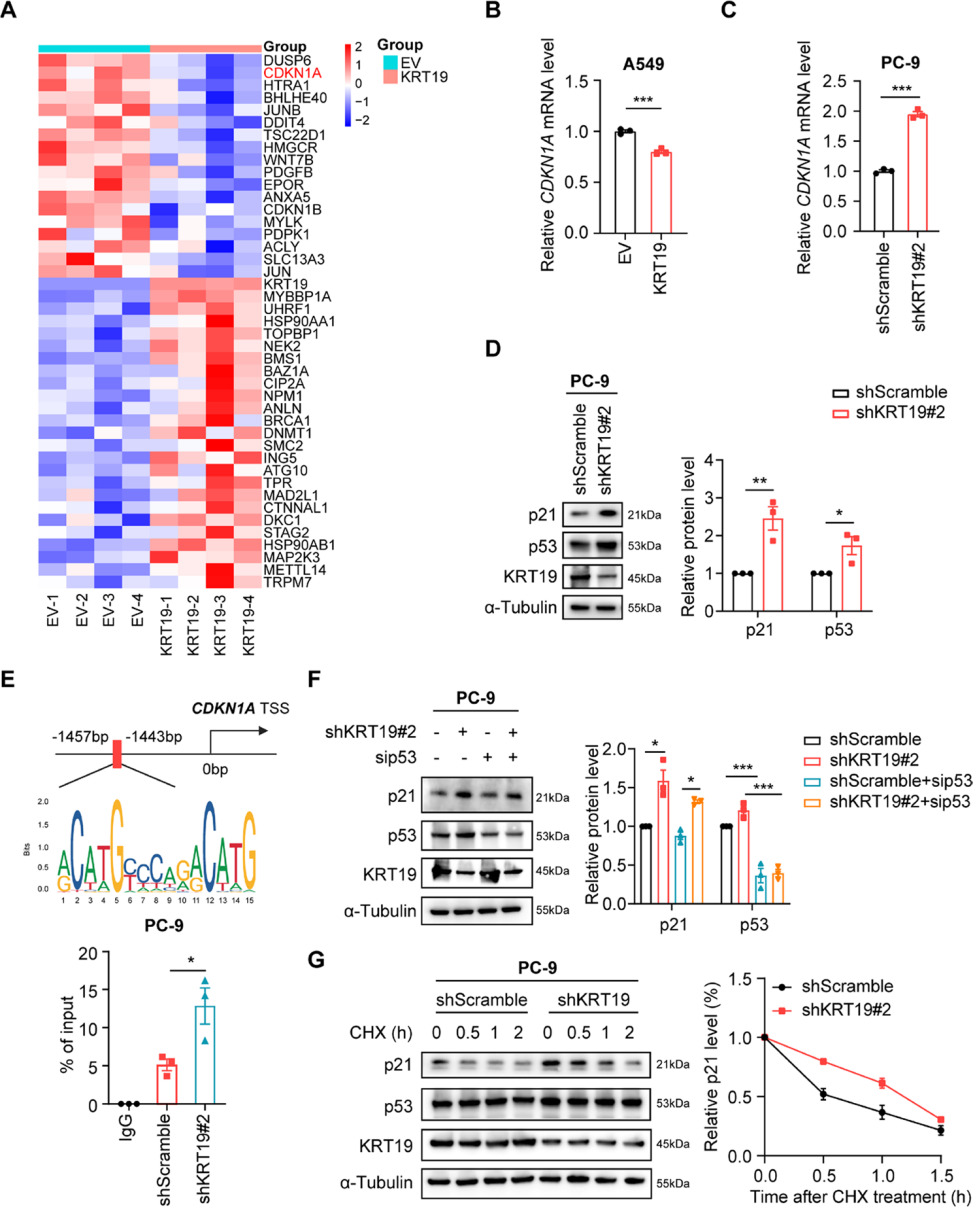
KRT19 reduces p53-dependent transcription of p21 and impairs p21 protein stability **A** Integrated analysis was performed between RNA-seq data of A549 cells (GSE298926) and the CellAge database. Heatmap visualizing differentially expressed genes related to cellular senescence was shown (*p* < 0.05) **B-C** *CDKN1A* mRNA expression in A549 (EV, KRT19, B) and PC-9 cells (shScramble, shKRT19#2, C) was measured by RT-PCR **D** The protein levels of p21, p53 and KRT19 in PC-9 cells (shScramble, shKRT19#2) were determined by immunoblot **E** The binding status of p53 in *CDKN1A* promoter region in PC-9 cells (shScramble, shKRT19#2) was measured by ChIP-PCR. TSS: transcription start site **F** PC-9 cells were transduced with shKRT19 lentivirus (shKRT19#2) or transfected with p53 siRNA (sip53) for 48 h. p21, p53 and KRT19 protein levels were measured by immunoblot **G** p21, p53 and KRT19 protein levels in PC-9 cells (shScramble, shKRT19#2) treated with cycloheximide (CHX, 15 µg/mL) for the indicated time were measured by immunoblot (left), and quantitation of p21 protein levels based on band intensity was shown (right). Data are shown as mean ± S.E.M. and analyzed by Student’s *t*-test (B-E) or one-way ANOVA (F). *p < 0.05; **p < 0.01; ***p < 0.001. The experiments (B-G) were repeated three times

To gain insights into the mechanism underlying the connection between KRT19 and NSCLC cell senescence, we jointly analyzed RNA-seq data (GEO accession number: GSE298926) and CellAge database [45] and noticed that overexpression of KRT19 significantly inhibited the level of the cell cycle inhibitor p21, the well-known gene associated with cell senescence (Fig. 5A-B). Meanwhile, we also found that knockdown of KRT19 remarkably upregulated the p21 mRNA and protein expression in PC-9 cells (Fig. 5C-D). It’s known that p53 transcriptionally activates p21, which plays important roles in cell growth arrest [46]. Immunoblot and ChIP-PCR showed that silencing of KRT19 led to an increased p53 level in NSCLC cells and significantly enhanced p53 binding to the *CDKN1A* promoter in PC-9 cells compared to controls (Fig. 5D-E). Interestingly, knockdown of KRT19 could still induce p21 expression in PC-9 cells bearing silencing of p53 (Fig. 5F), suggesting that KRT19 might also regulate p21 expression in a p53-independent pathway. Post-translational modification is crucial for protein expression. We found that KRT19 knockdown notably stabilized p21 but not p53 protein as compared to control (Fig. 5G), further suggesting that KRT19 could inhibit p21 expression by impairing its stability.

Altogether, these data demonstrated that KRT19 inhibits p21 expression in both p53-dependent and p53-independent manners.

### KRT19 interacts with MYH9 and induces its expression to facilitate p21 ubiquitination at K16

**Fig. 6:**
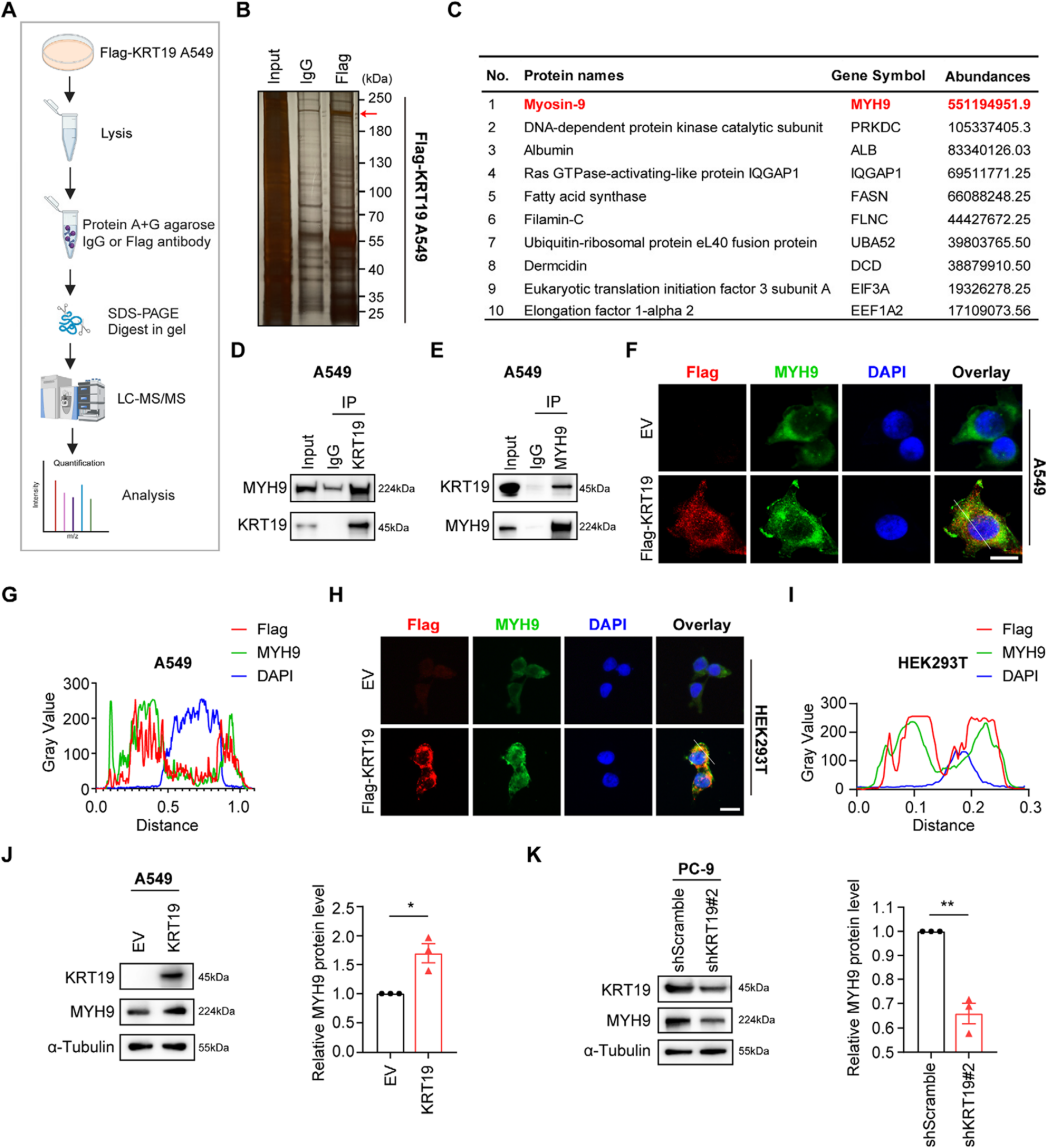
KRT19 interacts with MYH9 and induces its expression **A** Schematic representation of experimental setup for identifying KRT19-interacting proteins **B** Representative SDS-PAGE separation and silver staining of protein lysates from Flag-KRT19-overexpressing A549 cells immunoprecipitated with anti-Flag or control IgG **C** List of potential KRT19-binding proteins identified by mass spectrometry **D-E** The interaction of endogenous KRT19 and MYH9 in A549 cells was determined by Co-IP **F-I** A549 (F-G) and HEK293T cells (H-I) were transfected with MYH9-overexpressing plasmid and transduced with Flag-KRT19 lentivirus (Flag-KRT19 group) or control empty vector (EV group). Representative immunofluorescence images of A549 (F) and HEK293T cells (H), and co-localization analysis of Flag and MYH9 in Flag-KRT19 A549 (G) and HEK293T cells (I) were shown. Scale bar, 10 μm **J-K** KRT19 and MYH9 protein levels in different groups of A549 cells (J) and PC-9 cells (K) were measured by immunoblot. Data are shown as mean ± S.E.M. and analyzed by Student’s *t*-test (J-K). *p < 0.05; **p < 0.01. The experiments were repeated two (A-C) or three times (D-K)

To mechanistically clarify how KRT19 regulates p21 protein stability in NSCLC cells, we focused on the potential proteins interacted with KRT19 in NSCLC cells using immunoprecipitation (IP) coupled with mass spectrometry (Fig. 6A-C), among which MYH9 showed high affinity enrichment and has been found to be dysregulated and oncogenic in many cancers [47–50]. In the Co-IP experiments, we further confirmed KRT19 interacted with MYH9 in NSCLC cells (Fig. 6D-E). Consistently, we also detected the colocalization of KRT19 and MYH9 occurs abundantly in the cytoplasm using immunofluorescence (Fig. 6F-I). In addition, immunoblot analysis found that overexpression of KRT19 significantly upregulated the level of MYH9 while KRT19 knockdown led to reduced MYH9 expression in NSCLC cells (Fig. 6J-K). These data indicated that KRT19 interacts with MYH9 and induces its expression.

**Fig. 7:**
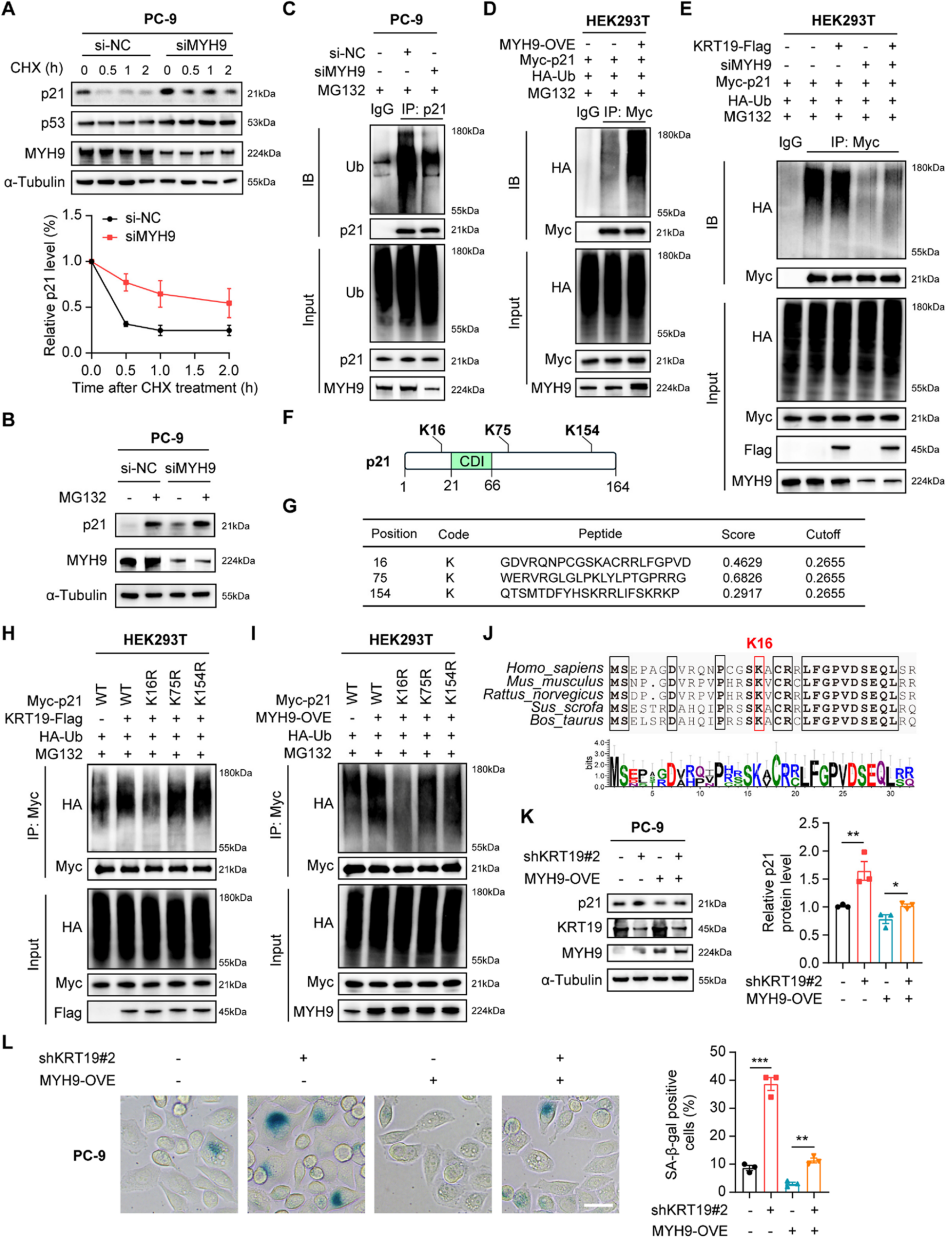
KRT19 facilitates p21 ubiquitination at K16 via MYH9 **A** PC-9 cells were transfected with MYH9 siRNA (siMYH9) or control siRNA (si-NC). The protein levels of p21, p53 and MYH9 in PC-9 cells (si-NC, siMYH9) treated with CHX (15 µg/mL) for the indicated time were measured by immunoblot (Top), and quantitation of p21 protein levels based on band intensity was shown (bottom) **B** p21 and MYH9 protein expression in PC-9 cells (si-NC, siMYH9) treated with or without MG132 (20 µM) for 4 h was determined by immunoblot **C** Co-IP analysis of the interaction between p21 and ubiquitin (Ub) in PC-9 cells (si-NC, siMYH9) treated with MG132 (20 µM) for 4 h **D** HEK293T cells were co-transfected with Myc-p21, HA-Ub and MYH9-overexpressing (MYH9-OVE) plasmids or control plasmid. Co-IP analysis of the interaction between Myc-p21 and HA-Ub in different groups of HEK293T cells treated with MG132 (20 µM) for 4 h **E** Control or Flag-KRT19-overexpressing (Flag-KRT19) HEK293T cells were co-transfected with siMYH9 or si-NC, Myc-p21 and HA-Ub plasmids. **F-G** Prediction of ubiquitination sites of p21 protein using GPS-Uber **H-I** KRT19-Flag-OVE (H) or MYH9-OVE (I) HEK293T cells were co-transfected with HA-Ub and Myc-p21 (WT, K16R, K75R, K154R). The interaction between Myc-p21 and HA-Ub was determined by Co-IP after treatment with MG132 (20 µM) for 4 h **J** Sequence comparison around the K16 residue (red) of the p21 homologue in different species **K-L** PC-9 cells were transduced with shKRT19#2 or transfected with MYH9-OVE plasmid for 48 h. p21, KRT19, MYH9 protein expression levels (K) and SA-β-gal staining (L) of different groups of PC-9 cells were shown. Scale bar, 10 μm. Data are shown as mean ± S.E.M. and analyzed by one-way ANOVA (K-L). *p < 0.05; **p < 0.01; ***p < 0.001. The experiments (A-E, H-I, K-L) were repeated three times

MYH9 could interfere protein stability in various types of cancers [51]. We have found that KRT19 could inhibit p21 expression by impairing its stability (Fig. 5G), to identify whether KRT19 induced p21 degradation via MYH9, we employed a cycloheximide (CHX) chasing assay and found that knockdown of MYH9 markedly promoted the stabilization of p21 protein (Fig. 7A). Immunoblot further showed that MG132 treatment significantly promoted p21 protein accumulation and partially abolished the elevated expression of p21 in PC-9 cells bearing knockdown of KRT19 or MYH9 (Fig. 7B, Figure S6A), suggesting that the regulation of p21 protein stability by KRT19/MYH9 axis relies on the ubiquitin-proteasome pathway. To be consistent, Co-IP assay discovered that either KRT19 or MYH9 knockdown significantly blocked the ubiquitination of p21, and overexpression of KRT19 or MYH9 produced the opposite results (Fig. 7C-D, Figure S6B-C). Moreover, silencing of MYH9 weakened the effect of KRT19 on the ubiquitination of p21 (Fig. 7E), further indicating that KRT19 regulates p21 protein stability through MYH9-mediated ubiquitin-proteasome degradation. In addition, we recognized K16, K75 and K154 of p21 as the potential ubiquitination sites using GPS-Uber (Fig. 7F-G). Exogenous Co-IP, multiple sequence alignment and WebLogo analysis indicated that K16 is a strictly conserved residue of p21, mutant of which could prompt the disassembly of ubiquitin and p21, while K75R and K154R mutant was ubiquitinated indistinguishably to that of wild-type protein (Fig. 7H-J). These findings demonstrated that KRT19 interacts with MYH9 to facilitate p21 ubiquitination at K16.

We next investigated the regulatory function of KRT19/MYH9 axis on cellular senescence. In NSCLC cells, MYH9 knockdown led to SA-β-gal accumulation and remarkably upregulated the mRNA expression of *CDKN2A*, *IL1B*,* IL6* and *CXCL8* in PC-9 cells (Figure S7A-B). Consistently, under hydrogen peroxide-induced senescence condition, MYH9-overexpressing A549 cells showed decreased SA-β-gal intensity and lower *CDKN2A*, *IL1B*,* IL6* and *CXCL8* mRNA levels compared with control group (Figure S7C-D). Furthermore, rescue experiments were performed by overexpressing MYH9 in KRT19-depleted PC-9 cells. Immunoblot and β-gal staining found that MYH9 overexpression partially reversed KRT19 knockdown-induced p21 upregulation and β-gal accumulation (Fig. 7K-L), implying that MYH9-mediated p21 ubiquitination contributes to KRT19 deficiency-induced NSCLC cellular senescence.

### Elevated KRT19 is positively linked with poor prognosis in NSCLC patients

**Fig. 8:**
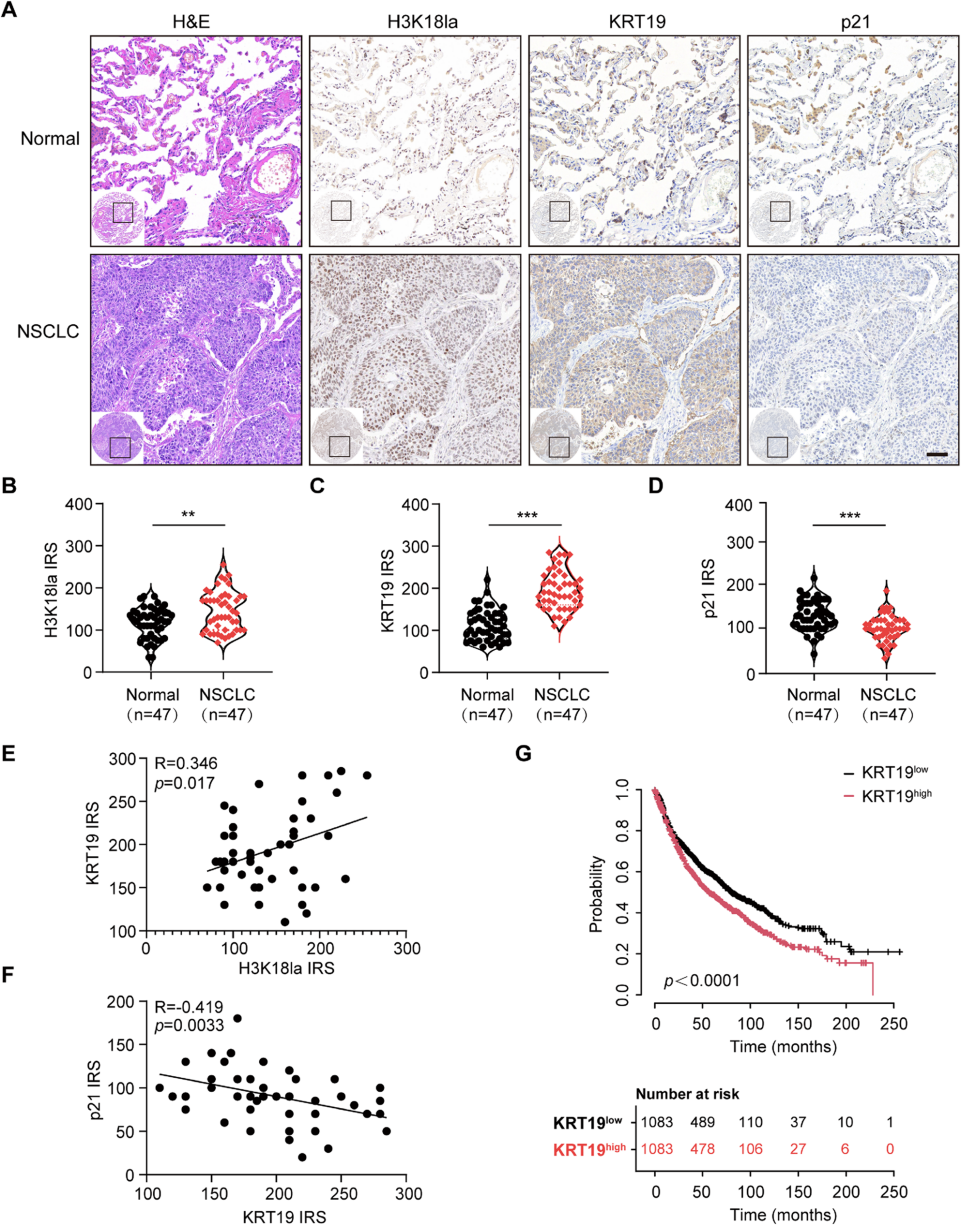
Elevated KRT19 is positively linked with poor prognosis in NSCLC patients **A-D** Representative H&E and IHC staining (A) and immunoreactive score (IRS) of H3K18la (B), KRT19 (C) and p21 (D) in clinical NSCLC tissues (*n* = 47) and normal control tissues (*n* = 47). Scale bar, 50 μm **E-F** Correlation between H3K18la and KRT19 (E), KRT19 and p21 (F) in NSCLC tissues (*n* = 47) **G** Kaplan-Meier survival analysis of NSCLC patients according to tumor KRT19 expression in Kaplan-Meier Plotter (Top). The number of NSCLC patients (KRT19^low^, KRT19^high^) at risk corresponding to each time point was shown (bottom). Data are shown as mean ± S.E.M. and analyzed by Student’s *t*-test (B-D). **p < 0.01; ***p < 0.001

We next identified the clinical significance of H3K18la/KRT19/p21 signaling in clinical NSCLC. Based on TCGA database, we found that NSCLC exhibited higher KRT19 expression than control group (Fig. 2B-C), and p21 levels were notably lower in NSCLC tissues compared with normal control (Figure S8). Then, we systematically identified the levels of H3K18la/KRT19/p21 by immunohistochemistry in a cohort of 47 NSCLC patients. Immunohistochemical analysis showed that H3K18la and KRT19 levels were significantly elevated, but p21 expression was decreased in clinical NSCLC specimens compared with adjacent normal tissues (Fig. 8A-D). Moreover, KRT19 expression was positively associated with H3K18la and negatively correlated to p21 expression levels in NSCLC tissues (Fig. 8E-F). In addition, we performed Kaplan-Meier survival analysis in Kaplan-Meier Plotter and illustrated that tumor KRT19 expression was positively correlated to poor prognosis in NSCLC patients, suggesting that KRT19 might serve as an indicator for overall survival of NSCLC patients (Fig. 8G).

These data further supported the notion that H3K18la/KRT19/p21 signaling plays a crucial role in tumor progression and prognosis of NSCLC patients.

### Silencing of KRT19 enhances anti-PD-1 immunotherapy efficiency in a NSCLC xenograft model

**Fig. 9:**
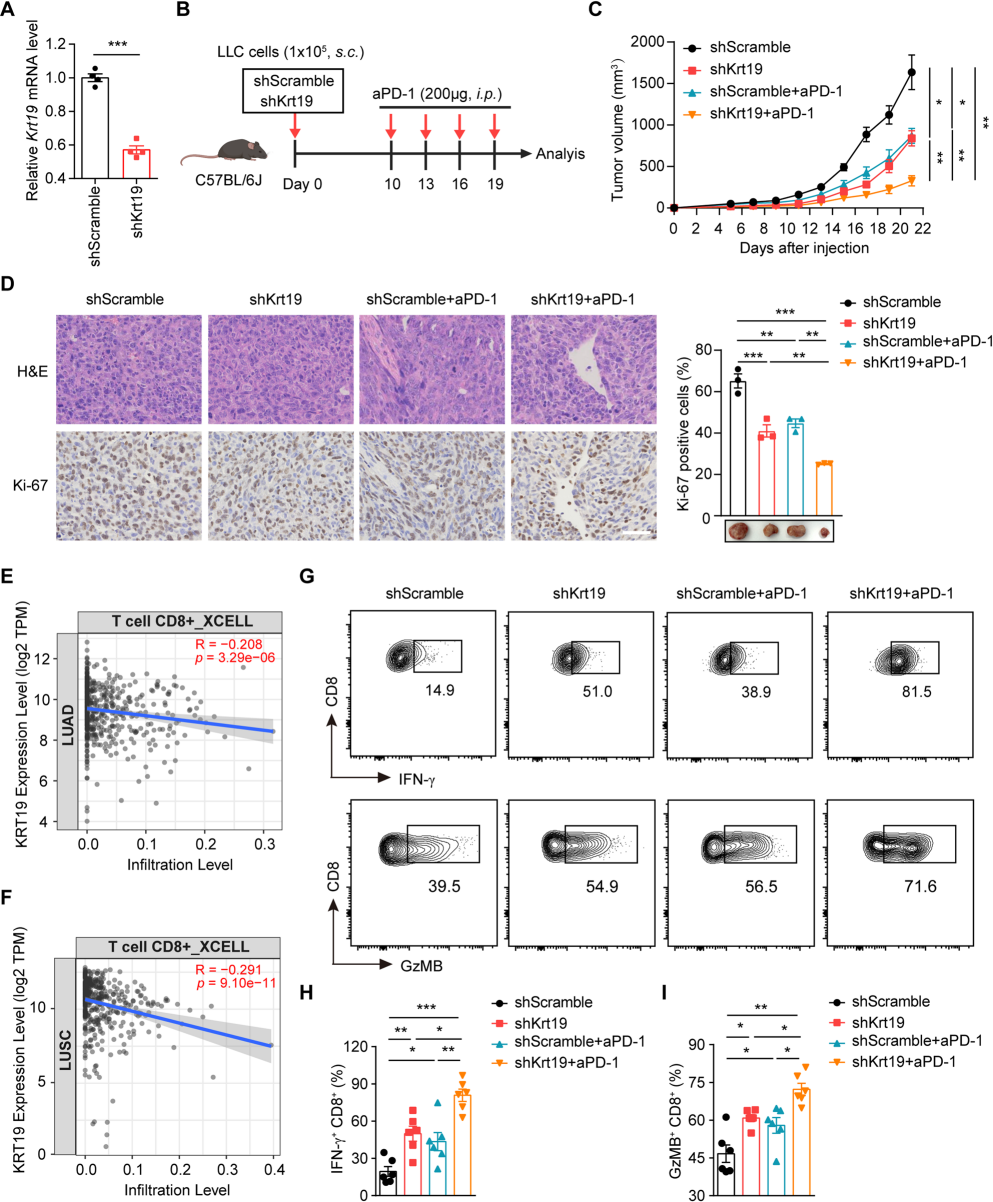
Silencing of KRT19 enhances anti-PD-1 immunotherapy efficiency in a NSCLC xenograft model **A** Stable knockdown of Krt19 in LLC cells was determined by RT-PCR **B-D** C57BL/6J mice (5 weeks of age, female) were subcutaneously (*s.c.*) injected with 1 × 10^5^ LLC cells. After 10 days, mice were intraperitoneally (*i.p.*) treated with anti-PD-1 (200 µg) or IgG every third day **B** Flowchart of in vivo experiment **C** Tumor growth curves were shown (*n* = 6) **D** IHC analysis of Ki-67-positive cells in LLC tumors (*n* = 3) and representative images of tumors subjected to various treatments were shown. Scale bar, 50 μm **E-F** Pearson correlation between immune infiltration level and KRT19 expression in lung adenocarcinoma (LUAD, E) and lung squamous cell carcinoma (LUSC, F) calculated by XCELL **G-I** Flow cytometric analysis of IFN-γ^+^ (G, H) and GzMB^+^ (G, I) CTLs isolated from tumor tissues within different groups (*n* = 6). Data are shown as mean ± S.E.M. and analyzed by Student’s *t*-test (A), two-way ANOVA (C) or one-way ANOVA (D, H-I). *p < 0.05; **p < 0.01; ***p < 0.001

Remodeling the TIME via the SASP is key to representing an emerging immunotherapeutic paradigm, which is critical to explore the next generation of cancer therapeutics [40]. Hypothesizing that SASP reprogramming with KRT19 knockdown in NSCLC cells might benefit anti-PD-1 immunotherapy, we stably silenced Krt19 using lentiviral particles in LLC cells and subcutaneously injected control or Krt19-knockdown LLC cells into the C57BL/6J mice, followed by treatment with anti-PD-1 (200 µg) or control IgG every three days (Fig. 9A-B). Combination of Krt19 knockdown in LLC and anti-PD-1 immunotherapy remarkably attenuated tumor growth compared with all other groups (Fig. 9C). Immunohistochemical analysis showed that the percentage of Ki-67-positive cells was significantly decreased in Krt19-knockdown xenografts treated with anti-PD-1 compared to therapeutic blockade of PD-1 alone (Fig. 9D).

To assess the TIME landscape reprogrammed by SASP, we firstly compared the infiltration of 22 immune cells between KRT19^high^ and KRT19^low^ NSCLC tissues obtained from TCGA database, and intratumoral CTLs infiltration was significantly restrained in NSCLC tissues with higher KRT19 expression (Figure S9A-B). XCELL analysis [52] further identified a negative correlation between intratumoral CTLs infiltration level and tumor KRT19 expression in NSCLC (Fig. 9E-F). Moreover, immunofluorescence assay also showed that tumor-infiltrating CTLs number was significantly higher in shKrt19 group compared with shScramble group (Figure S9C). Then, we investigated the effect of Krt19 inhibition and anti-PD-1 immunotherapy on tumor-infiltrating CTLs. Flow cytometric analysis showed that Krt19 knockdown in LLC promoted IFN-γ and Granzyme B (GzMB) levels in tumor-infiltrating CTLs, indicating the in vivo importance of KRT19-mediated cellular senescence in TIME reprogramming, while intervention of Krt19 in NSCLC and anti-PD-1 could synergistically enhance the IFN-γ and GzMB production in intratumoral CTLs (Fig. 9G-I).

In summary, these data verified that KRT19 knockdown enhances anti-PD-1 immunotherapy efficiency by potentiating stronger anti-tumor responses of tumor-infiltrating CTLs.

## Discussion

Cancer cells are metabolically heterogeneous and produce chromatin-modifying metabolites including lactate [53, 54]. In our study, we demonstrated that lactate directly accelerates NSCLC progression, blockade of which could remarkably induce senescence of NSCLC cells. Lactate-derived Kla, a newly identified PTM, mediates tumor progression through histone modification in various cancers [55–57]. For example, lactate has been acquainted to drive senescence-resistant hepatocellular carcinoma through lactylation of histone H2B at K58 on N-myc downstream-regulated gene 1 (NDRG1) [55]. In LUAD, LKB1 decreased histone H4 Lys8 and Lys16 lactylation to inhibit telomerase activity and thus induced cellular senescence [57]. Here, we detected increased H3K18la level in NSCLC, which activated downstream target KRT19 to resist senescence of NSCLC cells, further highlighting the significance of histone lactylation in NSCLC progression.

Upon p53 activation, cyclin-dependent kinase inhibitor p21 stimulates the retinoblastoma protein (RB) to inactivate E2F complexes, consequently resulting in cell cycle arrest [46]. Our study demonstrated that lactate-derived H3K18la transcriptionally activates KRT19 to inhibit p21 expression by downregulating p53, describing a novel regulatory mechanism of lactate in dictating the transcription of p21. *Zong* et al. discovered that alanyl-tRNA synthetase 1 (AARS1) mediated p53 lactylation at the K120 and K139 residues and hindered its liquid-liquid phase separation, DNA binding, and transcriptional activation of p21 [15], which is consistent with our notion that lactate is a natural inhibitor of the p53-p21 axis and senescence of tumor cells. On the other side, it becomes apparent that p21 could be regulated by alternative signaling pathways that are independent of p53. Ubiquilin 4 (Ubqln4) interacts with E3 ubiquitin ligase RING-type zinc-finger protein 114 (RNF114) to stabilize p21 by attenuating proteasomal degradation of p21 [58]. We revealed that KRT19 binds to MYH9 and induces its expression to facilitate ubiquitination and proteasomal degradation of the p21 without affecting p53 stability, establishing a novel epigenetic mechanism interlinking metabolic remodeling to cellular senescence.

Accumulating evidence has shown that KRT19 is abnormally expressed and correlates to poor prognosis in various cancers. Mechanistically, KRT19 regulates cancer cell properties, especially the reprogramming of cancer stem cells by mediating β-catenin [59], glycogen synthase kinase-3 (GSK3β) [21] et al. In the study herein, we found that KRT19 overexpression significantly prevents cellular senescence and exhibits oncogenic activity in NSCLC via MYH9-dependent ubiquitination of p21 at K16, revealing an unconventional role of KRT19 in dictating tumor cell fate. Cell-fate determination has evolved to be highly resilient to various perturbations [60]. In breast cancer, KRT19 is found to mediate cell cycle arrest, a typical characteristic of senescence by physically interacting with GSK3β and thus restrain GSK3β-dependent degradation of cyclin D3 [61]. MYH9 can also interact with GSK3β and facilitate GSK3β ubiquitination and degradation to favor cancer stemness properties in hepatocellular carcinoma [51], illustrating that KRT19/MYH9 may coordinate cell fate specification via orchestrating regulatory hubs with GSK3β. Comprehensive and integrated unraveling the intricate crosstalk and the dynamic molecular regulatory network of KRT19/MYH9 complex in dictating cell properties among different tumor types is of interest and needs further investigation.

During tumor initiation and progression, cellular senescence induces persistent cell cycle arrest, enhances immune clearance and finally avoids malignant expansion of cancer cells [62–65]. In the study herein, we found that tumor cell-intrinsic KRT19 deficiency results in cell cycle arrest in G1 phase and the accumulation of SA-β-gal, thus directly suppressing NSCLC progression ultimately. In addition, phenotypic shifts in immune cells regulated by cellular senescence can develop an immunosuppressive tumor microenvironment, which influences immune responses and treatment efficiency [66]. Therefore, specific induction of senescence in tumor cells, or the elimination of senescent cells by pharmacological interventions is gaining consideration in tumor treatment field [67–69]. Following the criterion, we identified KRT19 is highly expressed in NSCLC but not normal tissues, targeting of which not only induces NSCLC cell senescence, but also enhances the function of tumor-infiltrating CTLs and synergistically represses NSCLC progression when combining with anti-PD-1. Given that targeting senescent cells with senotherapies is postulated as a novel cancer treatment therapy, among which “one-two punch” strategy is intended to amplify the benefits of senescence induction and lessen related detrimental consequences [70]. In the future, high throughput screening and identifying KRT19 inhibitors and combining them with senotherapies might be a promising therapeutic strategy for the intervention of NSCLC and other tumors.

## Conclusions

In this study, we discovered that lactate-derived H3K18la is a critical factor for inducing the expression and oncogenic activity of KRT19, and intervention of KRT19 potently promotes p21-driven senescence program in NSCLC cells and boosts anti-PD-1 immunotherapy efficiency. Our study comprehensively characterizes the novel regulatory mechanism underlying H3K18la-driven KRT19 promotes NSCLC progression, yields promising insights into combination treatment strategies for patients with NSCLC.

## Supplementary Information

Supplementary Material 1.

## Data Availability

ChIP-seq (GSE245990) reported in this paper has been publicly available at the GEO database. RNA-seq datasets (GSE299060, GSE298926) generated in this work have been deposited at the GEO database and publicly available as of the date of publication. The data generated and analyzed during the current study are available from the corresponding author on reasonable request. Further information for reagents and resources (Table S3) will be available upon request from corresponding author.

## Abbreviations

AARS1: Alanyl-tRNA synthetase 1
CCL2: C-C motif chemokine ligand 2
CDKN1A: Cyclin dependent kinase inhibitor 1 A
ChIP-seq: Chromatin immunoprecipitation sequencing
CoREST: Corepressor of RE-1 silencing transcription factor
CTLs: Cytotoxic CD8^+^ T cells
CXCL1: C-X-C motif chemokine ligand 1
GSEA: Gene Set Enrichment Analysis
GSK3β: Glycogen synthase kinase-3β
GzMB: Granzyme B
HER2: Human epidermal growth factor receptor 2
HGF: Hepatocyte growth factor
H3K18la: H3K18 lactylation
IRS: Immunoreactive score
KEGG: Kyoto Encyclopedia of Genes and Genomes
Kla: Histone lysine lactylation
KRT19: Keratin 19
LDHA: Lactate dehydrogenase A
LDHB: Lactate dehydrogenase B
LKB1: Liver kinase B1
LLC: Lewis lung carcinoma
MYH9: Myosin heavy chain 9
NSCLC: Non-small cell lung cancer
NDRG1: N-myc downstream-regulated gene 1
NNMT: Nicotinamide N-methyltransferase
PTM: Post-translational modification
RB: Retinoblastoma protein
RBM4: RNA binding motif protein 4
RNA-seq: RNA-sequencing
RNF114: RING-type zinc-finger protein 114
SASP: Senescence-associated secretory phenotype
SA-β-gal: Senescence-associated β-galactosidase
SETD8: SET domain-containing protein 8
TIME: Tumor immune microenvironment
TCGA: The Cancer Genome Atlas Program
TGF-β: Transforming growth factor-β
Ub: Ubiquitin
Ubqln4: Ubiquilin 4
USP4: Ubiquitin-specific protease 4
