# Supplementary material for: Lactylation-driven KRT19 promotes non-small cell lung cancer progression by suppressing cellular senescence
Source: J Exp Clin Cancer Res. 2025 Dec 4;45:13. doi: 10.1186/s13046-025-03602-5 (PMC12802155; doi:10.1186/s13046-025-03602-5)
Supplement: Supplementary file 1 — Supplementary Material 1. [file 13046_2025_3602_MOESM1_ESM.docx]

**Supplemental information**

**Lactylation-driven KRT19 promotes non-small cell lung cancer progression by suppressing cellular senescence**

Cai Zhang, Yue Du, Yangyang Ji, Xiaoxiao Ye, Jingyao Lian, Haonan Zhou, Zihan Gao, Huiping Xu, Yuehan Tang, Yanhong Fan, Lu Zheng

**
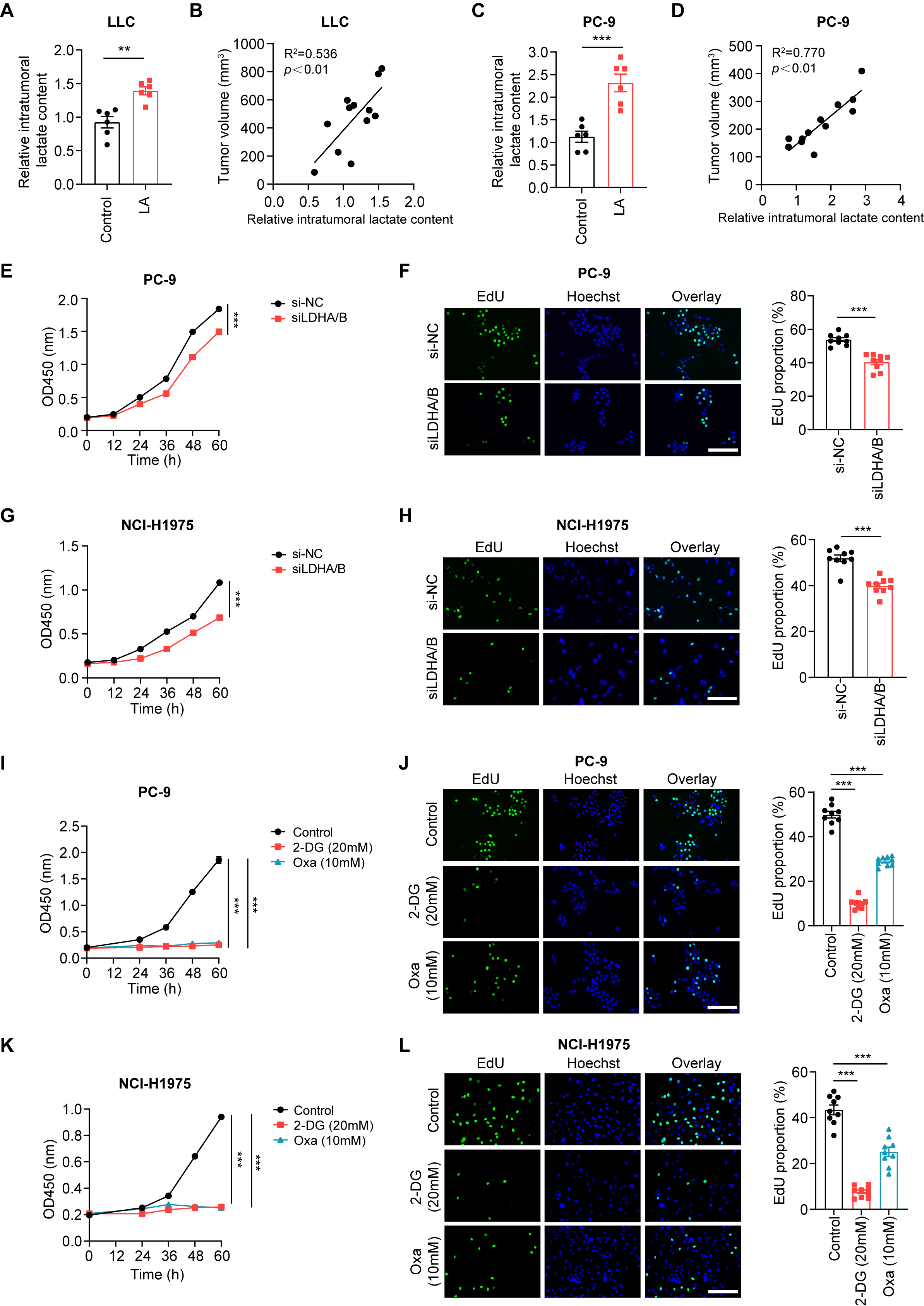
**

**Figure S1. Blocking cell-intrinsic lactate production prevents NSCLC cell growth, related to Figure 1.**

(**A**-**D**) BALB/c-nude mice (5-6 weeks of age, female) were subcutaneously injected with 1×10^6^ LLC or 1×10^6^ PC-9 cells and intratumorally treated with either lactate (40 μL of 40 mM, LA group) or saline (Control group) when the tumor tissues reached 50 mm^3^. Lactate content in control and lactate-treated LLC tumors (A, n=6) and PC-9 tumors (C, n=6) were measured. Correlation analysis of intratumoral lactate content with tumor volume of LLC (B) and PC-9 tumors (D) was shown. (**E-H**) PC-9 (E-F) and NCI-H1975 cells (G-H) were transfected with siLDHA and siLDHB (siLDHA/B group) or control siRNA (si-NC group). (**E, G**) Cell viability of PC-9 (E) and NCI-H1975 cells (G) in si-NC or siLDHA/B group was determined by CCK8 assay. (**F, H**) Proliferation of PC-9 (F) and NCI-H1975 cells (H) transfected with siLDHA and siLDHB (siLDHA/B group) or control siRNA (si-NC group) was measured by EdU assay. (**I-L**) PC-9 (I-J) and NCI-H1975 cells (K-L) were incubated with 2-DG (20 mM, 2-DG group), oxamate (10 mM, Oxa group) or vehicle (Control group) for indicated time. (**I, K**) Cell viability of PC-9 (I) and NCI-H1975 cells (K) after stimulation with 2-DG (20 mM), oxamate (10 mM) or vehicle for indicated time was determined by CCK8 assay. (**J, L**) PC-9 and NCI-H1975 cells were treated with 2-DG (20 mM), oxamate (10 mM) or vehicle for 24 h. Representative images and quantification of EdU-positive PC-9 (J) and NCI-H1975 cells (L) in different groups were shown. For the EdU assay (F, H, J, L), Green, EdU; blue, Hoechst. Scale bar, 50 μm (3 technical replicate wells, 3 fields per well). Data are shown as mean ± S.E.M. and analyzed by Student’s *t*-test. ***p* < 0.01; ****p* < 0.001. The experiments (E-L) were repeated three times.

**
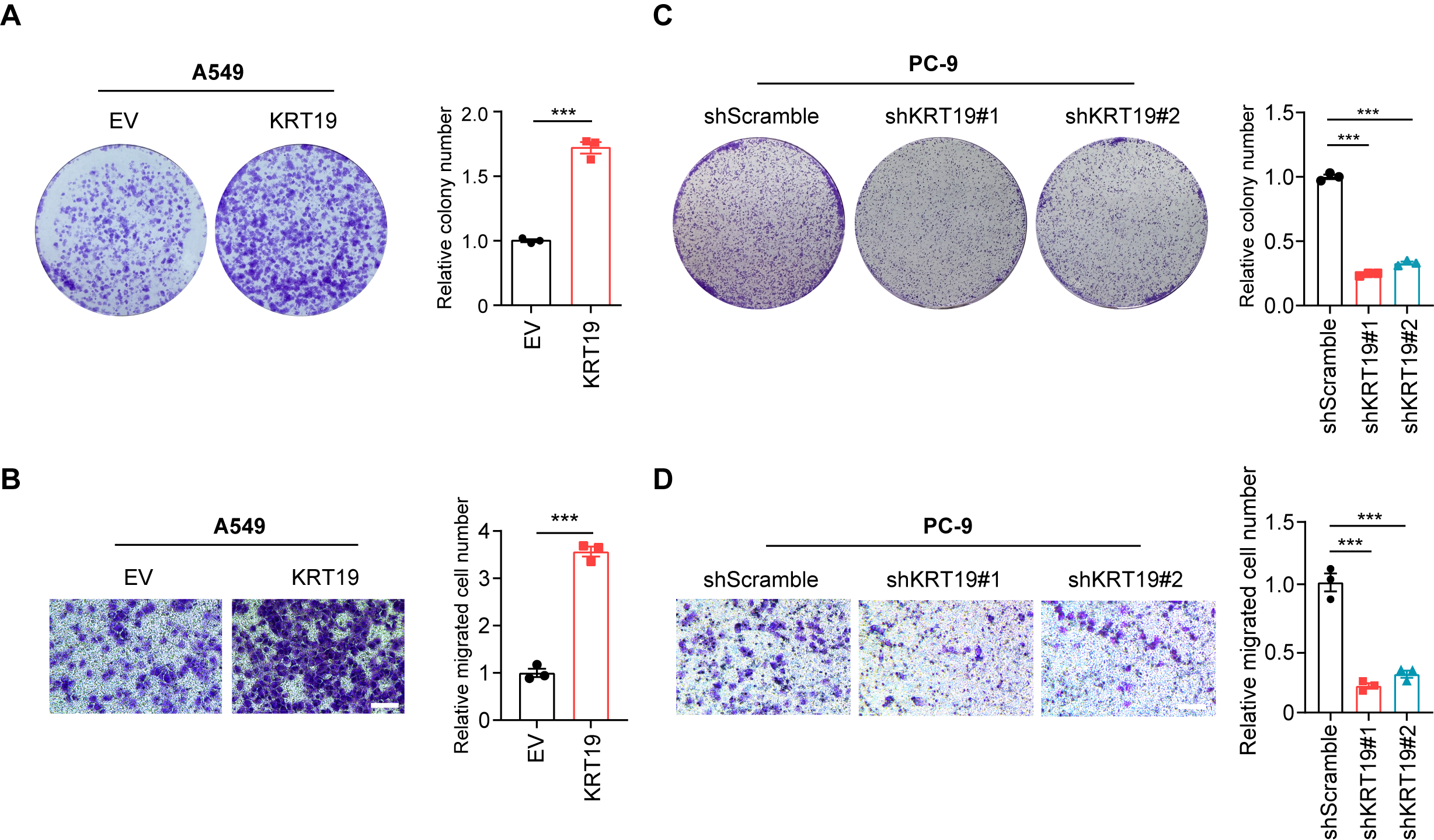
**

**Figure S2. KRT19 promotes the cluster formation and migration of NSCLC cells, related to Figure 2.**

**(A-B**) A549 cells were transduced with KRT19-overexpressing lentiviral vector (KRT19 group) or control empty vector (EV group). The cluster formation and migration of A549 cells (EV, KRT19) were determined by the colony formation assay (A) and transwell assay (B), respectively. Scale bar, 20 μm. (**C-D**) PC-9 cells were transduced with scramble shRNA lentivirus (shScramble) or KRT19 shRNA lentivirus (shKRT19#1, shKRT19#2). The cluster formation and migration of PC-9 cells (shScramble, shKRT19#1, shKRT19#2) were determined by the colony formation assay (C) and transwell assay (D), respectively. Scale bar, 20 μm. Data are shown as mean ± S.E.M. and analyzed by Student’s *t*-test. ****p* < 0.001. The experiments were repeated three times.

**
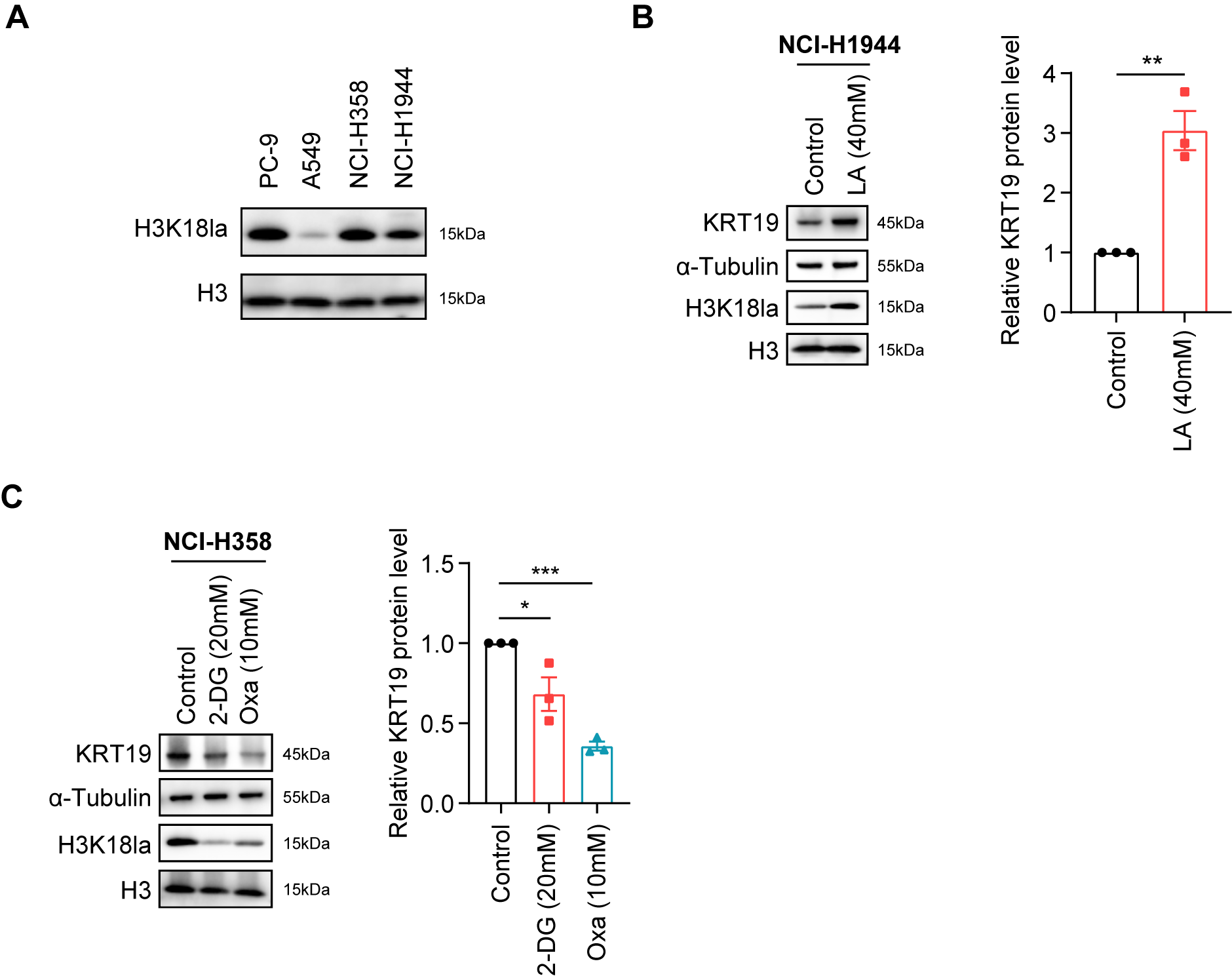
**

**Figure S3. Lactate-derived H3K18la positively regulates KRT19 expression in NSCLC cells, related to Figure 3.**

(**A**) H3K18la protein expression in NSCLC cell lines (PC-9, A549, NCI-H358, NCI-H1944) was determined by immunoblot. (**B**) H3K18la and KRT19 protein in NCI-H1944 cells incubated with lactate (40 mM, LA group) or vehicle (Control group) for 24 h. (**C**) H3K18la and KRT19 protein expression levels in NCI-H358 cells treated with 2-DG (20 mM, 2-DG group), oxamate (10 mM, Oxa group) or vehicle (Control group) for 24 h. Data are shown as mean ± S.E.M. and analyzed by Student’s *t*-test. **p* < 0.05; ***p* < 0.01; ****p* < 0.001. The experiments were repeated three times.

**
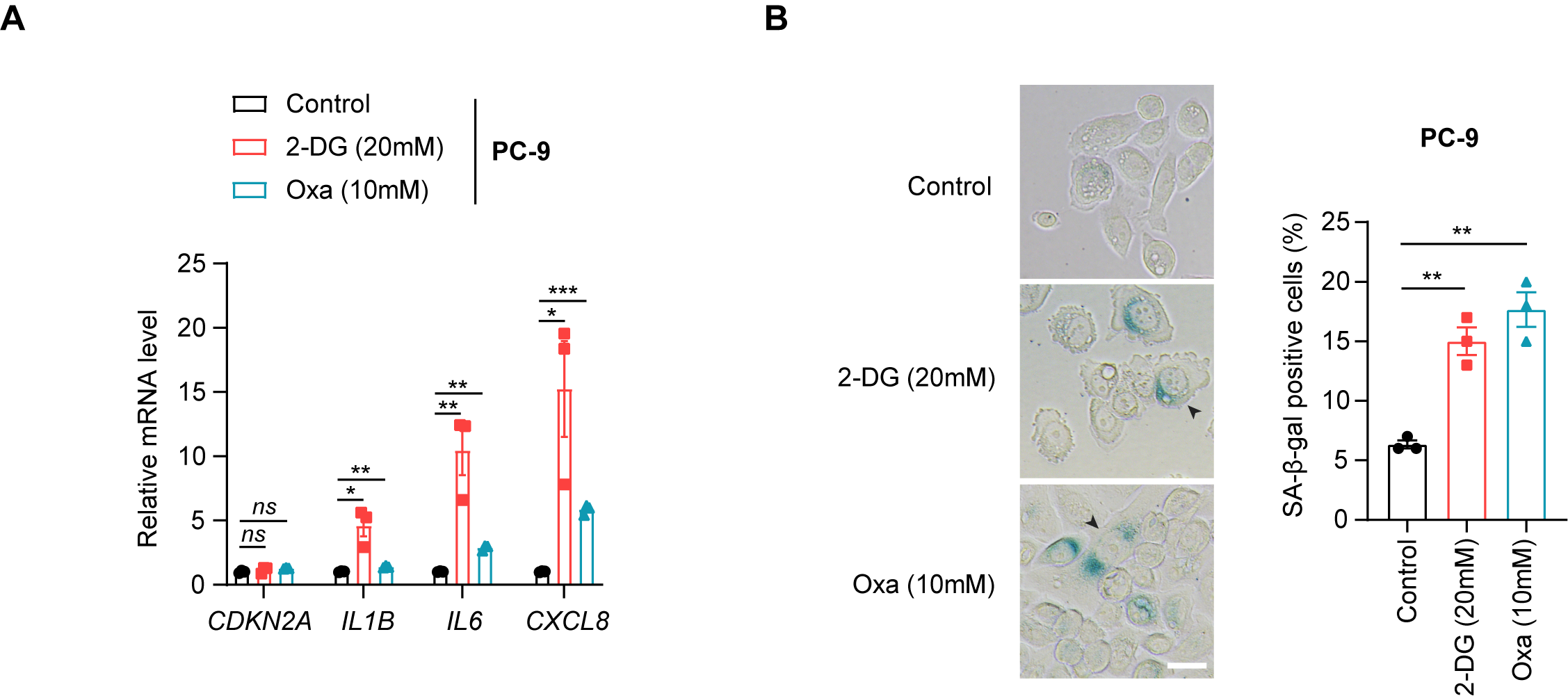
Figure S4. Glycolysis inhibition induces cellular senescence of PC-9 cells, related to Figure 4.**

PC-9 cells were treated with 2-DG (20 mM, 2-DG group), oxamate (10 mM, Oxa group) or vehicle (Control group) for 24 h. (**A**) *CDKN2A, IL1B, IL6* and *CXCL8* mRNA expression in PC-9 cells subjected to various treatments was measured by RT-PCR. (**B**) SA-β-gal staining of PC-9 cells (Control, 2-DG, Oxa). The arrow indicates the enlarged morphologic changes. Scale bar, 10 μm. Data are shown as mean ± S.E.M. and analyzed by Student’s *t*-test. **p* < 0.05; ***p* < 0.01; ****p* < 0.001; ns, not significant. The experiments were repeated three times.

**
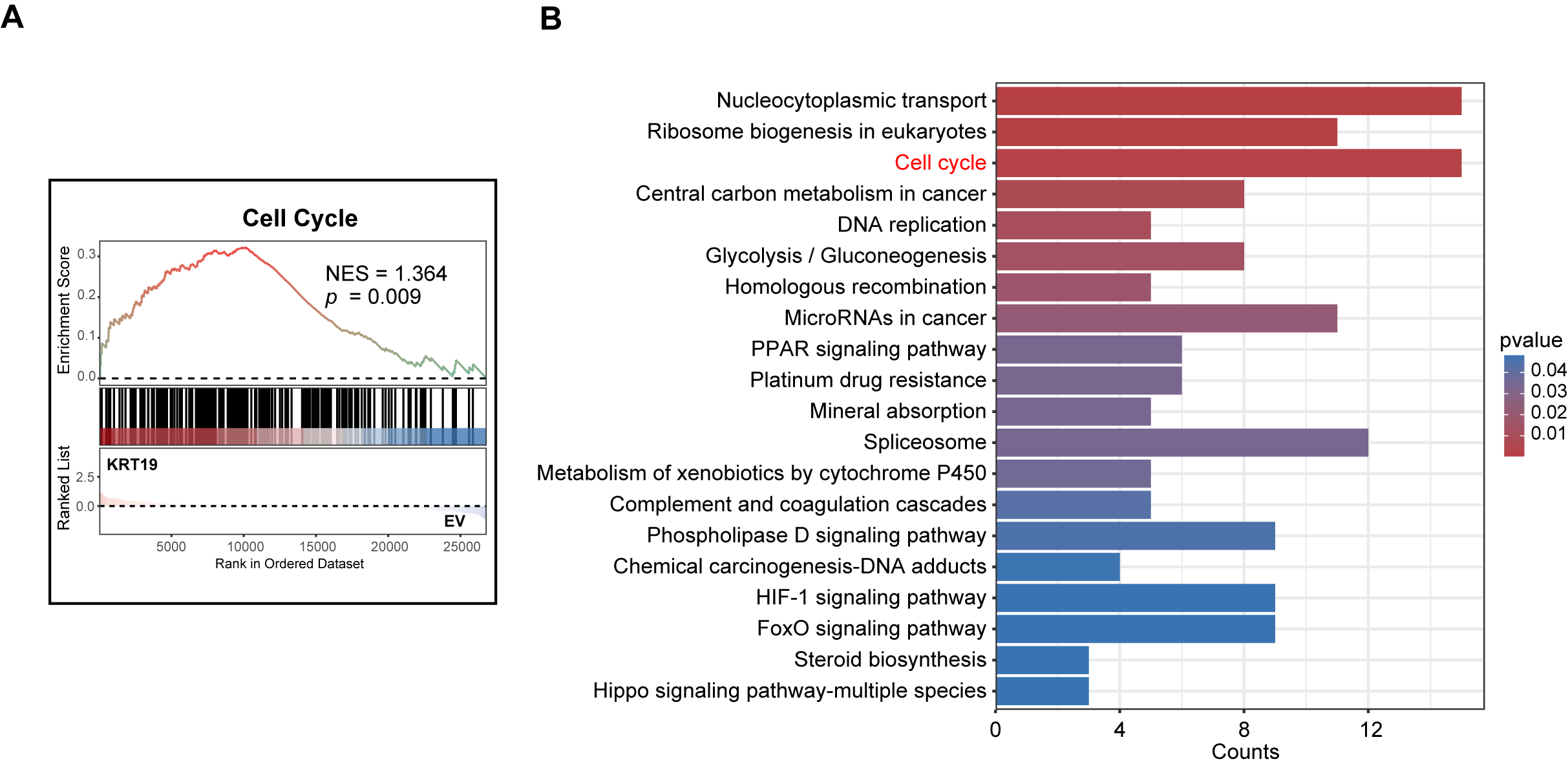
**

**Figure S5. KRT19 overexpression activates cell cycle signaling in A549 cells, related to Figure 4.**

(**A-B**) RNA-seq (GSE298926) was performed in A549 cells transduced with KRT19-overexpressing lentiviral vector (KRT19 group) or control empty vector (EV group). (A) Gene set enrichment analysis (GSEA) plot evaluating cell cycle signal in A549 cells (EV, KRT19). (B) Top 20 KEGG pathways enriched by differentially expressed genes from RNA-seq data between KRT19 group and EV group (*p* < 0.05).

**
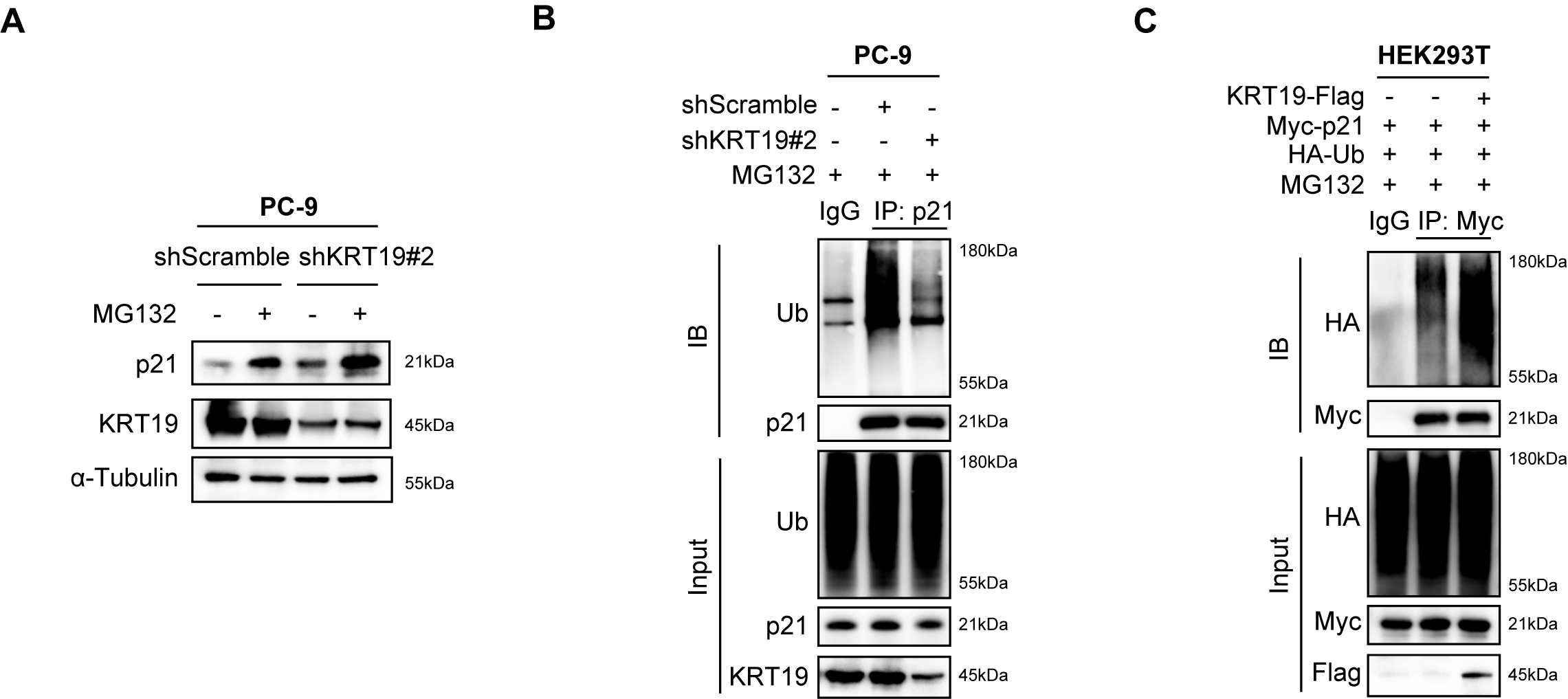
**

**Figure S6. KRT19 facilitates p21 ubiquitination, related to Figure 7.**

(**A**) PC-9 cells were transduced with scramble shRNA lentivirus (shScramble) or KRT19 shRNA lentivirus (shKRT19#2). p21 and KRT19 protein expression in PC-9 cells (shScramble, shKRT19#2) treated with or without MG132 (20 μM) for 4 h was determined by immunoblot. (**B**) Co-IP analysis of the interaction between p21 and ubiquitin (Ub) in PC-9 cells (shScramble, shKRT19#2) treated with MG132 (20 μM) for 4 h. (**C**) HEK293T cells were co-transfected with Myc-p21 and HA-Ub plasmids in control or Flag-KRT19-overexpressing (Flag-KRT19) HEK293T cells. The interaction between Myc-p21 and HA-Ub was determined by Co-IP in different groups of HEK293T cells treated with MG132 (20 μM) for 4 h. The experiments were repeated three times.

**
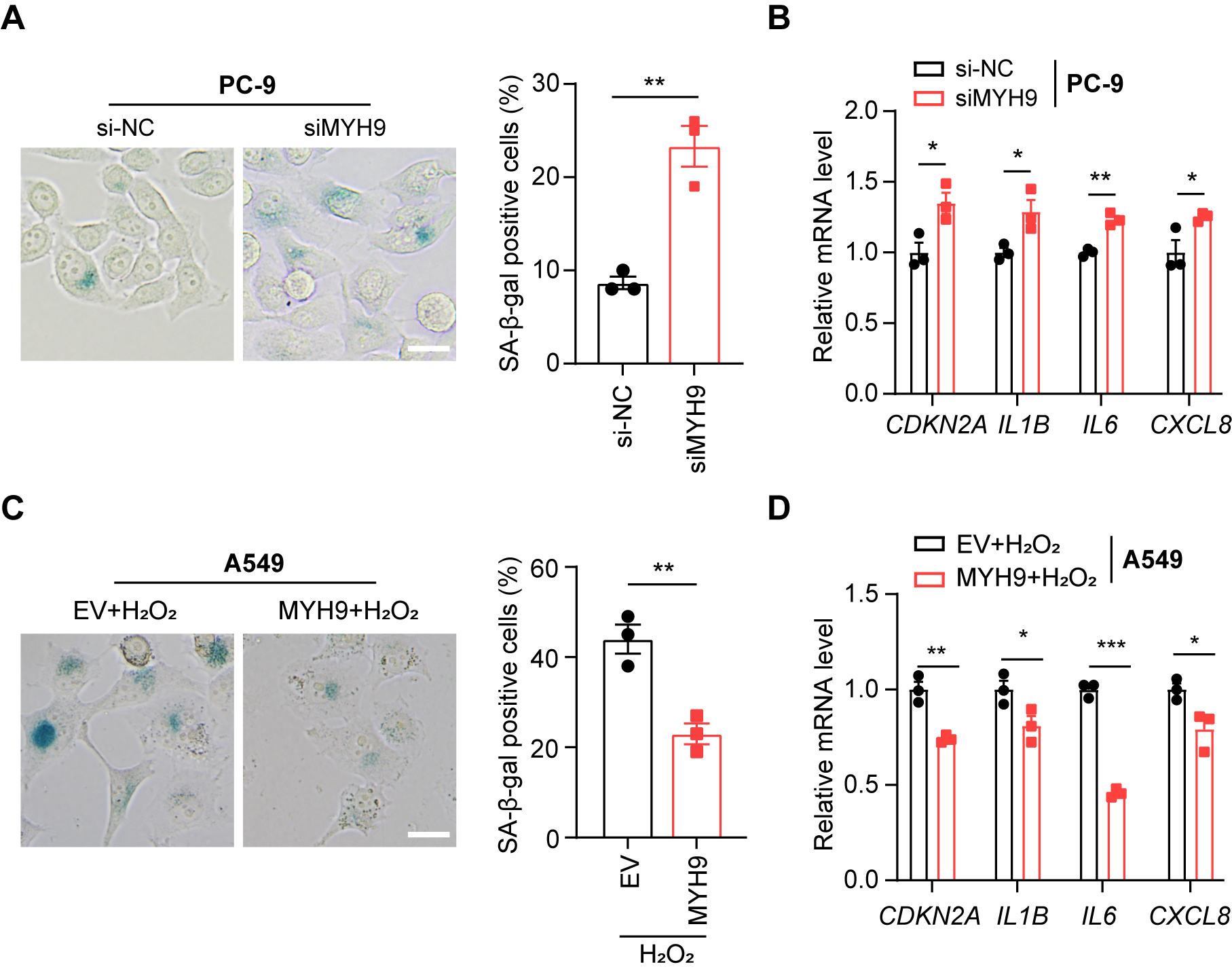
**

**Figure S7. MYH9 suppresses senescence in NSCLC cells, related to Figure 7.**

(**A**-**B**) PC-9 cells were transfected with MYH9 siRNA (siMYH9) or control siRNA (si-NC). (**A**) SA-β-gal staining of PC-9 cells (si-NC, siMYH9). Scale bar, 10 μm. (**B**) *CDKN2A, IL1B, IL6* and *CXCL8* mRNA expression in PC-9 cells (si-NC, siMYH9) was measured by RT-PCR. (**C**) A549 cells transfected with MYH9-overexpressing plasmid (MYH9 group) or empty vector (EV group) were treated with hydrogen peroxide (H_2_O_2_, 50 μM) for 24 h. SA-β-gal staining of A549 cells was shown. Scale bar, 10 μm. (**D**) A549 cells transfected with MYH9-overexpressing plasmid (MYH9 group) or empty vector (EV group) were treated with hydrogen peroxide (H_2_O_2_, 100 μM) for 24 h. *CDKN2A, IL1B, IL6* and *CXCL8* mRNA levels in A549 cells were determined by RT-PCR. Data are shown as mean ± S.E.M. and analyzed by Student’s *t*-test. **p* < 0.05; ***p* < 0.01; ****p* < 0.001. The experiments were repeated three times.

**
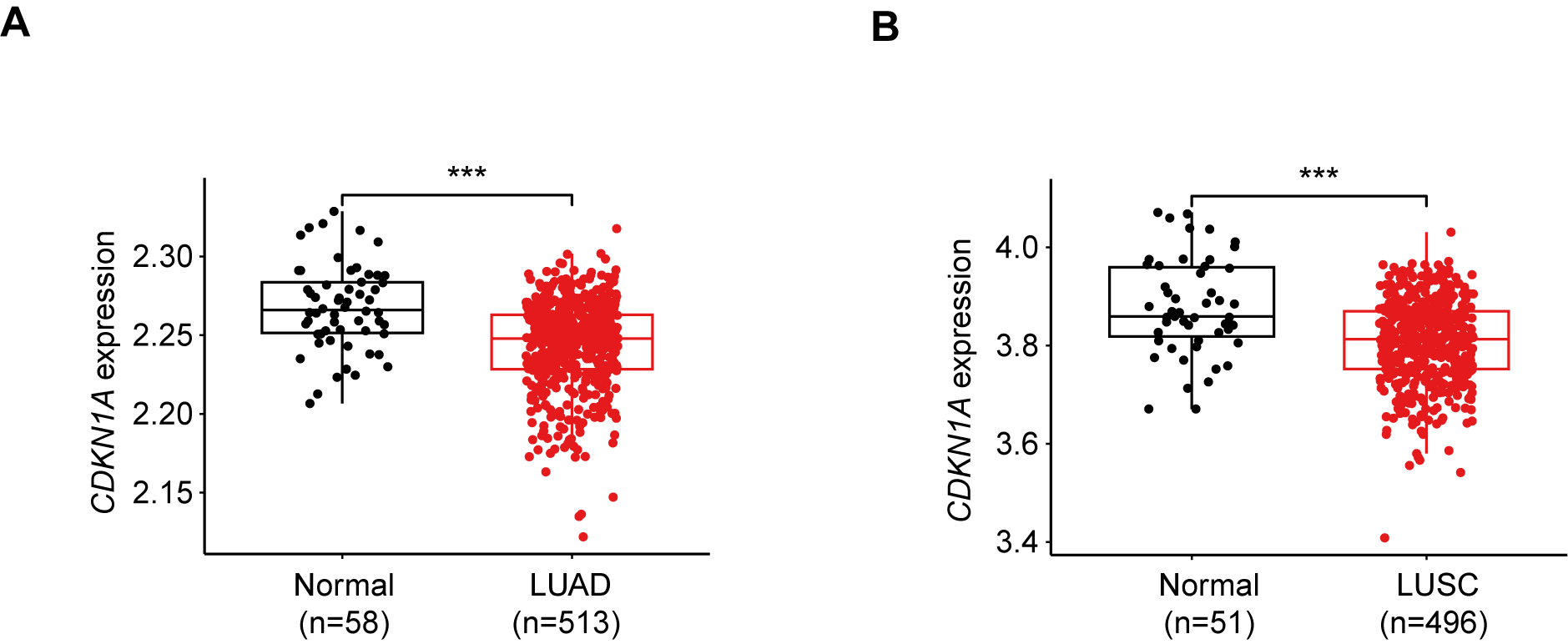
**

**Figure S8. *CDKN1A* mRNA expression is elevated in LUAD and LUSC, related to Figure 8.**

(**A-B**) *CDKN1A* mRNA expression levels in LUAD (A) and LUSC (B) samples obtained from The Cancer Genome Atlas (TCGA) database were analyzed using R software (version 4.3.1). *** *p*<0.001.

**
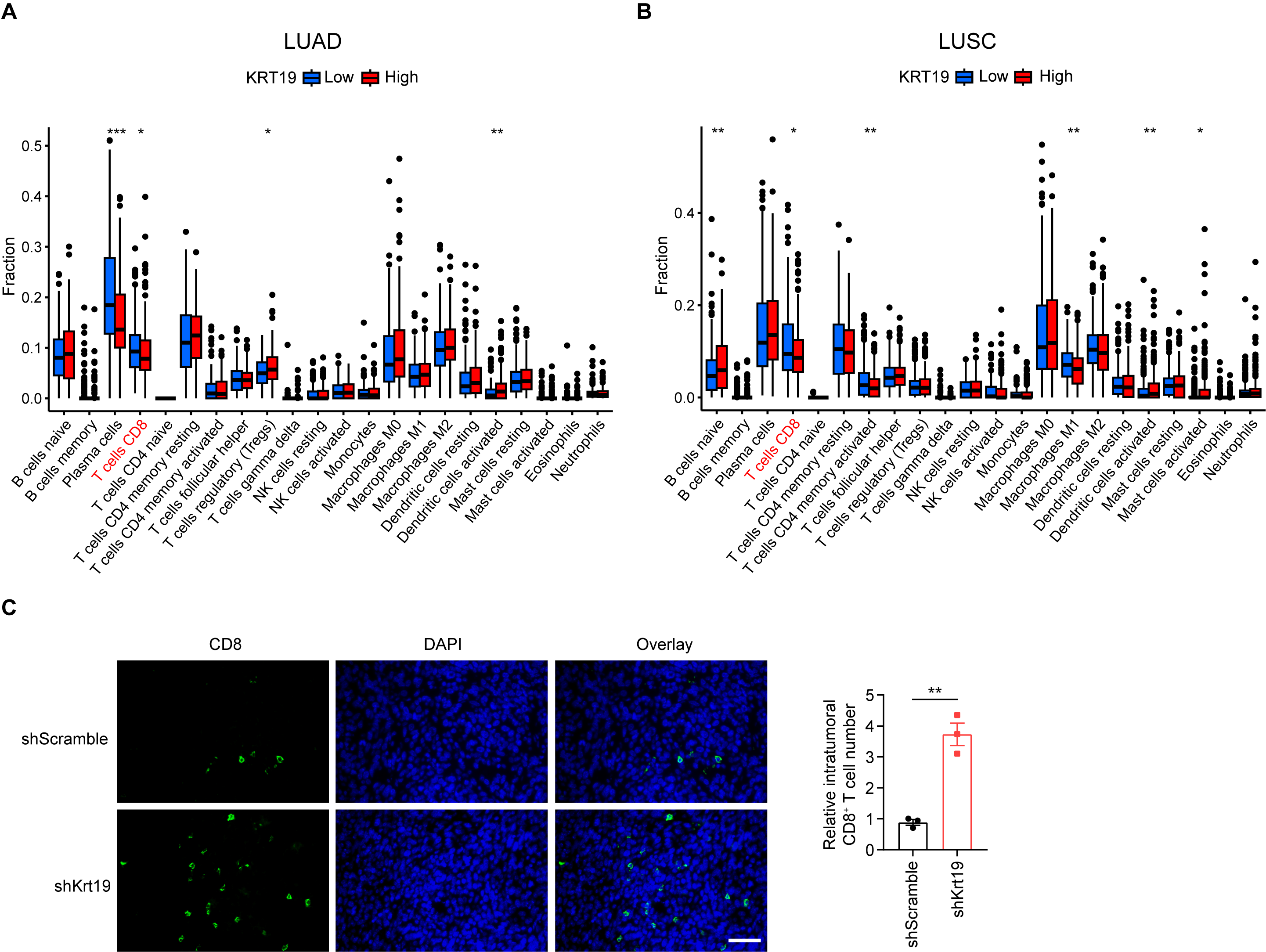
**

**Figure S9. KRT19 expression was negatively correlated with intratumoral CD8^+^ T cell infiltration in NSCLC, related to Figure 9.**

**(A-B)** Comparative analysis of the infiltration levels of 22 immune cells in KRT19^high^ and KRT19^low^ LUAD (A) and LUSC (B) tissues. Data were obtained from the TCGA database and analyzed using CIBERSORT. (**C**) C57BL/6J mice (5 weeks of age, female) were subcutaneously injected with 1×10^5^ LLC cells (shScramble, shKrt19) as illustrated in Fig. 8. Immunofluorescence analysis of tumor-infiltrating CD8^+^ T cell number in LLC tumors (shScramble, shKrt19, n=3). Scale bar, 50 μm. Data are shown as mean ± S.E.M. and analyzed by Student’s *t*-test (C). **p* < 0.05; ***p* < 0.01; *** *p* < 0.001.

**Table S1.** Primers sequences

| **Gene** | **Sequence (5'->3')** |
| --- | --- |
| *Krt19* (Forward) | GGTTCAGTACGCATTGGGTC |
| *Krt19* (Reverse) | TAAAACTTCCACCGCGGACT |
| *Actb* (Forward) | GGCTGTATTCCCCTCCATCG |
| *Actb* (Reverse) | CCAGTTGGTAACAATGCCATGT |
| *KRT19* (Forward) | TGAGTGACATGCGAAGCCAA |
| *KRT19* (Reverse) | TCAGTAACCTCGGACCTGCT |
| *CDKN2A* (Forward) | GCCTTTTCACTGTGTTGGAG |
| *CDKN2A* (Reverse) | TGCCATTTGCTAGCAGTGTG |
| *CDKN1A* (Forward) | TGTCCGTCAGAACCCATGC |
| *CDKN1A* (Reverse) | AAAGTCGAAGTTCCATCGCTC |
| *IL1B* (Forward) | TTCGACACATGGGATAACGACG |
| *IL1B* (Reverse) | TTTTTGCTCTGAGTCCCGGAG |
| *IL6* (Forward) | CCTGAACCTTCCAAAGATGGC |
| *IL6* (Reverse) | TTCACCAGGCAAGTCTCCTCA |
| *CXCL8* (Forward) | ATGACTTCCAAGCTGGCCGTG |
| *CXCL8* (Reverse) | TGTGTTGGCGCAGTGTGGTC |
| *ACTB* (Forward) | TGGCACCACACCTTCTACAAT |
| *ACTB* (Reverse) | GATAGCACAGCCTGGATAGCA |
| *KRT19* promoter-a (Forward) | TGTGTAGGAATATGACCCCA |
| *KRT19* promoter-a (Reverse) | CTCTCTCTGCAGACCCTGTT |
| *KRT19* promoter-b (Forward) | AATCGCAGCTTCTGAGACCA |
| *KRT19* promoter-b (Reverse) | ACGAGGACACAAAGCGGGC |
| *CDKN1A* promoter (Forward) | TGCCTCATGAGGACTCAGCA |
| *CDKN1A* promoter (Reverse) | TGGCACCTACCTGCCTGCTCT |
| p21 K16R (Forward) | ATGCGGCAGCAGGGCCTGCCGCCGCCTCTT |
| p21 K16R (Reverse) | CGGCAGGCCCTGCTGCCGCATGGGTTCTGA |
| p21 K75R (Forward) | TTGGCCTGCCCAGGCTCTACCTTCCCA |
| p21 K75R (Reverse) | AAGGTAGAGCCTGGGCAGGCCAAGGCC |
| p21 K154R (Forward) | ACCACTCCAGACGCCGGCTGATCTTCTCCAA |
| p21 K154R (Reverse) | AGCCGGCGTCTGGAGTGGTAGAAATCTGTCA |

| **Reagent** | **Sequence (5'->3')** | **Source** |
| --- | --- | --- |
| Human  LDHA siRNA | Sense: GGCAAAGACUAUAAUGUAA/dT//dT/ | Sangon |
|  | Antisense: UUACAUUAUAGUCUUUGCC/dT//dT/ |  |
|  |  |  |
| Human  LDHB siRNA | Sense: GGUGCAGAGAAAUGUUAAU/dT//dT/ | Sangon |
|  | Antisense: AUUAACAUUUCUCUGCACC/dT//dT/ |  |
|  |  |  |
| Human  p53 siRNA | Sense: GUCUUUGAACCCUUGCUUGTT | GenePharma |
|  | Antisense: CAAGCAAGGGUUCAAAGACTT |  |
|  |  |  |
| Human  MYH9 siRNA | Sense: GCAAAUUCAUUCGCAUCAAdTdT | GenePharma |
|  | Antisense: AUACUUAUCGGCAGCUUGCdTdT |  |
|  |  |  |
| Negative control siRNA | Sense: UUCUUCGAACGUGUCACGUTT | GenePharma |
|  | Antisense: ACGUGACACGUUCGGAGAATT |  |

**Table S2.** siRNA targeting sequences

| **Table S3.** Reagents and resources |  |  |
| --- | --- | --- |
| **REAGENT or RESOURCE** | **SOURCE** | **IDENTIFIER** |
| **Antibodies** | | |
| Rabbit monoclonal anti-CDK1 (EPR165) | Abcam | Cat#ab133327;  RRID: AB_11155333 |
| Mouse monoclonal anti-LDHB (431.1) | Santa Cruz | Cat#sc-100775;  RRID: AB_1124720 |
| Mouse monoclonal anti-Ubiquitin (A-5) | Santa Cruz | Cat#sc-166553;  RRID: AB_2241297 |
| Normal mouse IgG | Santa Cruz | Cat#sc-2025;  RRID: AB_737182 |
| Rabbit monoclonal anti-L-Lactyl-Histone H3 (Lys18) | PTM BIO | Cat#PTM-1406RM;  RRID: AB_2909438 |
| Rabbit monoclonal ChIP Grade anti-L-Lactyl-Histone H3 (Lys18) | PTM BIO | Cat#PTM-1427RM;  RRID: AB_307669 |
| Rabbit polyclonal anti-Cytokeratin 19 | Proteintech | Cat#10712-1-AP;  RRID: AB_2133325 |
| Rabbit polyclonal anti-p21 | Proteintech | Cat#10355-1-AP;  RRID: AB_2077682 |
| Rabbit polyclonal anti-p53 | Proteintech | Cat#10442-1-AP;  RRID: AB_2206609 |
| Rabbit polyclonal anti-MYH9 | Proteintech | Cat#11128-1-AP;  RRID: AB_2147294 |
| Rabbit polyclonal anti-Histone H3 | Proteintech | Cat#17168-1-AP;  RRID: AB_2716755 |
| Mouse monoclonal anti-Alpha Tubulin | Proteintech | Cat#66031-1-Ig;  RRID: AB_11042766 |
| Mouse monoclonal anti-Flag tag | Proteintech | Cat#66008-4-Ig;  RRID: AB_2918475 |
| Rabbit polyclonal anti-HA tag | Proteintech | Cat#51064-2-AP;  RRID: AB_11042321 |
| HRP-conjugated IgG Fraction Mouse Anti-Rabbit IgG, Light Chain Specific | Proteintech | Cat#SA00001-7L;  RRID: AB_2890988 |
| HRP-conjugated Mouse Anti-Heavy Chain of Rabbit IgG | Proteintech | Cat#SA00001-7H;  RRID: AB_2935611 |
| Normal rabbit IgG | Beyotime | Cat#A7016;  RRID: AB_2905533 |
| HRP Goat Anti Rabbit IgG (H+L) | ImmunoWay | Cat#RS0002;  RRID: AB_2943496 |
| HRP Goat Anti Mouse IgG (H+L) | ImmunoWay | Cat#RS0001;  RRID: AB_2943495 |
| Rabbit monoclonal anti-Cyclin D1 | Abways | Cat#CY5404;  RRID: AB_3676731 |
| Rabbit monoclonal anti-CDK6 | Abways | Cat#CY5835;  RRID: AB_3697418 |
| Rabbit monoclonal anti-LDHA | Abways | Cat#CY5348;  RRID: AB_3697419 |
| Mouse monoclonal anti-Myc tag | Abways | Cat#AB0001;  RRID: AB_3697420 |
| Goat Anti-Rabbit IgG (AF488) | Abways | Cat#AB0141 |
| Goat Anti-Mouse IgG (AF594) | Abways | Cat#AB0152 |
| Rabbit recombinant anti-CD8 alpha | Servicebio | Cat#GB15068;  RRID: AB_3246431 |
| Rabbit polyclonal anti-Ki67 | Servicebio | Cat#GB111499;  RRID: AB_2927572 |
| Anti-mouse CD3-PerCP | BioLegend | Cat#100325;  RRID: AB_893319 |
| Anti-mouse CD8a-APC | BioLegend | Cat#100711;  RRID: AB_312750 |
| Anti-human/mouse Granzyme B-PE | BioLegend | Cat#372207;  RRID: AB_2687031 |
| Anti-mouse IFN-γ-FITC | BioLegend | Cat#505806;  RRID: AB_315400 |
| Invivo mouse IgG1 isotype control | Starter | Cat#S0B0788 |
| Invivo anti-mouse PD-1 Recombinant mAb | Starter | Cat#S0B0594 |
| **Chemicals, peptides, and recombinant proteins** | | |
| DMEM medium | BasalMedia | Cat#L110KJ |
| RPMI-1640 medium | BasalMedia | Cat#L220KJ |
| Fetal bovine serum | BasalMedia | Cat#S660JY |
| Penicillin/streptomycin | NCM Biotech | Cat#C100C5 |
| Puromycin | Solarbio | Cat#P8230 |
| TRLIP DNA Transfection Reagent | Kemix | Cat#KD0201 |
| RNATransMate | Sangon | Cat#E607402 |
| TRIzol | CWBIO | Cat#CW0580S |
| 2×SYBR Green qPCR Premix | Kermey | Cat#MS0601 |
| All-in-One Script RTpremix | Kermey | Cat#MR0502 |
| Collagenase IV | Thermo Fisher Scientific | Cat#17104019 |
| DNase I | Roche | Cat#10104159001 |
| Percoll gradient | Cytiva | Cat#17089101 |
| Cell Stimulation Cocktail | BioLegend | Cat#423303 |
| Cytofix/Cytoperm Solution | BD | Cat#554715 |
| Propidium iodide | Solarbio | Cat#C0080 |
| RNase A | Solarbio | Cat#R1030 |
| Phosphoprotease Inhibitor | Servicebio | Cat#G2007 |
| PMSF | Servicebio | Cat#G2008 |
| RIPA Lysis Buffer | Servicebio | Cat#G2002 |
| IP Lysis Buffer | Servicebio | Cat#G2038 |
| Bovine serum albumin | Kermey | Cat#MB0101 |
| DAPI | Kermey | Cat#MD0139 |
| Triton X-100 | Servicebio | Cat#G3068 |
| Protein A+G Agarose | Beyotime | Cat#P0255 |
| 4% Paraformaldehyde | Servicebio | Cat#G1101 |
| Crystal violet | Servicebio | Cat#G1014 |
| Lactate | Sigma-Aldrich | Cat#L7022 |
| 2-Deoxy-D-glucose (2-DG) | APExBIO | Cat#B1027 |
| Oxamate | MCE | Cat#HY-W013032A |
| Cycloheximide | GlpBio | Cat#GC17198 |
| MG132 | GlpBio | Cat#GC10383 |
| Proteinase K | Beyotime | Cat#ST533 |
| Hieff Mut™ Targeted Mutagenesis Kit | Yeasen | Cat#11003ES10 |
| DLR Assay System | Promega | Cat#E1910 |
| Cell Counting Kit-8 | GlpBio | Cat#GK10001 |
| BeyoClick™ EdU Cell Proliferation Kit with AF488 | Beyotime | Cat#C0071S |
| ChIP Assay Kit | Beyotime | Cat#P2078 |
| Omni-ECL Kit | Epizyme | Cat#SQ201 |
| SA-β-Gal Staining Kit | Beyotime | Cat#C0602 |
| Fast Silver Stain Kit | Beyotime | Cat#P0017S |
| BCA Protein Assay Kit  Lactic Acid Assay Kit | NCM Biotech  Solarbio | Cat#WB6501  Cat#BC5340 |
| **Experimental models** | | |
| PC-9 | Cell Bank/Stem Cell Bank, Chinese Academy of Sciences | Cat#SCSP-5085 |
| A549 | Cell Bank/Stem Cell Bank, Chinese Academy of Sciences | Cat#SCSP-503 |
| NCI-H358 | Cell Bank/Stem Cell Bank, Chinese Academy of Sciences | Cat#SCSP-583 |
| NCI-H1944 | Cell Bank/Stem Cell Bank, Chinese Academy of Sciences | Cat#SCSP-596 |
| NCI-H1299 | Cell Bank/Stem Cell Bank, Chinese Academy of Sciences | Cat#SCSP-589 |
| NCI-H1975 | Servicebio | Cat#STCC10204P |
| LLC | Servicebio | Cat#STCC20006P |
| HEK293T | Servicebio | Cat#STCC10301 |
| BALB/c-nude mice | Huafukang | Cat#13001A |
| C57BL/6J mice | Huafukang | Cat#11001A |
| Tissue microarray | Outdo Biotech |  |
| **Recombinant DNA** | | |
| HA-Ubiquitin | Addgene | Cat#18712 |
| pCMV-EGFP-MYH9 | Miaoling Bio | Cat#P53699 |
| pEnCMV-CDKN1A-3xMyc | Miaoling Bio | Cat#P30729 |
| pEnCMV-CDKN1A-3xMyc -K16R | This paper | N/A |
| pEnCMV-CDKN1A-3xMyc -K75R | This paper | N/A |
| pEnCMV-CDKN1A-3xMyc -K154R | This paper | N/A |
| pRL-TK | Promega | Cat#E2241 |
| pGL3-basic vector | Promega | Cat#E1751 |
| pGL3-*KRT19* (-448 bp to 163 bp) | This paper | N/A |
